# Supplementary material for: Cross-sectional investigation of household transmission of Cryptosporidium in England and Wales: the epiCrypt study protocol
Source: BMJ Open. 2019 Jun 21;9(6):e026116. doi: 10.1136/bmjopen-2018-026116 (PMC6596955; doi:10.1136/bmjopen-2018-026116)
Supplement: Author's manuscript [file bmjopen-2018-026116.draft_revisions.pdf]

BMJ Open

BMJ Open is committed to open peer review. As part of this commitment we make the peer review history of every article we publish publicly available.

When an article is published we post the peer reviewers' comments and the authors' responses online. We also post the versions of the paper that were used during peer review. These are the versions that the peer review comments apply to.

The versions of the paper that follow are the versions that were submitted during the peer review process. They are not the versions of record or the final published versions. They should not be cited or distributed as the published version of this manuscript.

BMJ Open is an open access journal and the full, final, typeset and author-corrected version of record of the manuscript is available on our site with no access controls, subscription charges or pay-per-view fees (http://bmjopen.bmj.com).

If you have any questions on BMJ Open's open peer review process please email info.bmjopen@bmj.com

BMJ Open

The epiCrypt study protocol: a cross-sectional investigation of household transmission of Cryptosporidium in England and Wales

Journal:	*BMJ Open*
Manuscript ID	bmjopen-2018-026116
Article Type:	Protocol
Date Submitted by the Author:	29-Aug-2018

Complete List of Authors:	McKerr, Caoimhe; university of liverpool, NIHR Health Protection Research Unit in Gastrointestinal Infections; University of Liverpool, NIHR Health Protection Research Unit in Emerging and Zoonotic Infections<br>Chalmers, Rachel; Public Health Wales, Cryptosporidium Reference Unit; Swansea University Medical School<br>Vivancos, Roberto; Public Health England, Field Epidemiology Services, Health Protection; The University of Liverpool, NIHR Health Protection Research Unit in Emerging and Zoonotic Infections<br>O'Brien, Sarah; University of Liverpool, Institute of Psychology, Health and Society; University of Liverpool, NIHR Health Protection Research Unit in Gastrointestinal Infections<br>Mugarza, Julie; NIHR Clinical Research Network North West Coast<br>Christley, Robert; University of Liverpool, Department of Epidemiology and Population Health, Institute of Infection and Global Health; NIHR Health Protection Research Unit in Emerging and Zoonotic Infections

Keywords:	Cryptosporidium, Household transmission, Secondary spread, Zoonoses, Gastrointestinal infection, Epidemiology < TROPICAL MEDICINE

SCHOLARONE™
Manuscripts

Short title

The epiCrypt Study - protocol

Word Count

≈3700 (excl. figs, tbls, refs)

Authors:

Caoimhe McKerr[1,2,6] / c.mckerr@liv.ac.uk

Rachel M Chalmers[3,7] / rachel.chalmers@wales.nhs.uk

Roberto Vivancos[1,2,4] / roberto.vivancos@phe.gov.uk

Sarah J O'Brien[1] / sjobrien@liv.ac.uk

Julie Mugarza[5] / julie.mugarza@nihr.ac.uk

Robert M Christley[2,6] / robc@liv.ac.uk

1 NIHR Health Protection Research Unit in Gastrointestinal Infections, The University of Liverpool

2 NIHR Health Protection Research Unit in Emerging and Zoonotic Infections, The University of Liverpool

3 *Cryptosporidium* Reference Unit, Public Health Wales, Swansea

4 Field Epidemiology Services, Public Health England, Liverpool

5 NIHR Clinical Research Network (CRN) North West Coast, Liverpool

6 Institute of Infection and Global Health, University of Liverpool

7 Swansea Medical School, Swansea University

For peer review only

Corresponding author:

Caoimhe McKerr

Current address:

The University of Liverpool

Institute of Infection and Global Health

Waterhouse Building (2nd Floor, Block F)

1-5 Brownlow Street

Liverpool L69 3GL

Email: c.mckerr@liverpool.ac.uk

For peer review only

TITLE

The epiCrypt study protocol: a cross-sectional investigation of household transmission of *Cryptosporidium* in England and Wales

AUTHORS

Caoimhe McKerr[1,2,6] / c.mckerr@liv.ac.uk

Rachel M Chalmers[3,7] / rachel.chalmers@wales.nhs.uk

Roberto Vivancos[1,2,4] / roberto.vivancos@phe.gov.uk

Sarah J O'Brien[1] / sjobrien@liv.ac.uk

Julie Mugarza[5] / julie.mugarza@nihr.ac.uk

Robert M Christley[2,6] / robc@liv.ac.uk

1 NIHR Health Protection Research Unit in Gastrointestinal Infections, The University of Liverpool

2 NIHR Health Protection Research Unit in Emerging and Zoonotic Infections, The University of Liverpool

3 *Cryptosporidium* Reference Unit, Public Health Wales, Swansea

4 Field Epidemiology Services, Public Health England, Liverpool

5 NIHR Clinical Research Network (CRN) North West Coast, Liverpool

6 Institute of Infection and Global Health, University of Liverpool

7 Swansea Medical School, Swansea University

Correspondence to: c.mckerr@liv.ac.uk

For peer review only

ABSTRACT

Introduction

Infection with the *Cryptosporidium* parasite causes over 4,000 cases of diagnosed illness (cryptosporidiosis) in England and Wales each year. Risk factors are often estimated from outbreak investigations, and in the UK include ingestion of contaminated water and food, farm/animal contact and person-to-person spread in institutions. However, reported outbreaks only represent about 10% of cases and the transmission routes for sporadic disease may not be the same. Contact with other people has been highlighted as a factor in the transmission of *Cryptosporidium*, but the incidence of sporadic disease has not been sufficiently established, and how frequently this arises from contact with other infected people is not well documented.

This project will estimate the amount of secondary spread that occurs in the home and potentially identify asymptomatic infections which might have a role in transmission. Risk factors and characteristics associated with secondary spread will be described including any differences in transmission between *Cryptosporidium* species.

Methods and analysis

The study will prospectively identify cryptosporidiosis cases from North West England and Wales over one year and invite them and their household to take part. Each household will complete a questionnaire and each household member will be asked to provide a stool sample. Clinical, demographic, and home variables will be described, and further analyses undertaken to investigate associations with secondary spread in the home.

Cryptosporidium-positive stool samples, identified by immunofluorescence microscopy, will be characterised using molecular methods to describe patterns of transmission. Data collection is expected to take one year, beginning in September 2018.

Ethics

The study has been approved by the North West – Liverpool East NHS Research Ethics Committee (Reference: 18/NW/0300) and the Confidentiality and Advisory Group (Reference

For peer review only

18/CAG/0084). The project is registered on the National Institute for Health Research (NIHR) portfolio (CPMS ID: 39458).

KEYWORDS

Cryptosporidium – Protozoa – Sporadic disease – Household transmission – Person-to-person – Secondary spread - Zoonoses – Gastrointestinal infection – Risk factors – Epidemiology - Parasite – Foodborne diseases – Waterborne diseases

STRENGTHS AND LIMITATIONS OF THIS STUDY

- This prospective household study will provide detailed information on the incidence of, and risk factors for, secondary spread of cryptosporidiosis in the home
- This study will characterise *Cryptosporidium* isolates to ascertain likely mechanisms of spread by species
- This study will potentially identify the prevalence of asymptomatic infections with *Cryptosporidium*
- Common exposures across households may present problems with accurately identifying true secondary spread
- Biases may lead to a skewed sample of index cases because of under-ascertainment and bias in health-seeking behaviours

For peer review only - http://bmjopen.bmj.com/site/about/guidelines.xhtml

INTRODUCTION

Cryptosporidium is a protozoan parasite which can infect humans and other animals, and the most prevalent species identified in humans are *Cryptosporidium parvum* and *Cryptosporidium hominis* [1,2]. *Cryptosporidium* is an important cause of diarrhoeal disease in the UK. Data from Public Health England (PHE) indicates that, on average, they receive laboratory reports of over 4,000 cases per year (mean 4,181 cases, 2000-2012 data) in England & Wales, but some research indicates that undiagnosed infections may place this closer to 64,000 cases per year (range 45,000-88,000) [3,4].

The parasite has a complex life cycle, and characteristics which favour faecal-oral and environmental transmission routes, which may facilitate outbreaks via person-to-person (*C. hominis and C. parvum*) or animal-to-person (*C. parvum*) contact, as well as indirect transmission through ingestion of water and food contaminated with infectious oocysts [5].

Risk factors and associated exposures are often hypothesised/identified from outbreak investigations, however outbreaks may only represent a small proportion of cases; estimates in the UK suggest, of all cases reported to national surveillance, <10% are likely to be linked to an identified outbreak [6]. Contact with other people is highlighted as a factor in the transmission of *Cryptosporidium* [7–10]. Studies of *Giardia*, another gastrointestinal parasite, similar in terms of likely transmission routes, have recently been undertaken in the UK, and secondary spread and person-to-person transmission seems a likely and under-quantified route of transmission [11,12].

Previous work

Several papers suggest that secondary spread is of significance to *Cryptosporidium* transmission, usually mediated by person-to-person contact, but how much this contributes to true sporadic disease has not been sufficiently established, and how much household spread follows-on from outbreaks is not well documented. In a 1988 paper, onward transmission of *Cryptosporidium* was reported in households following a nursery outbreak,

For peer review only

probably propagated by person-to-person spread in the home [7]. An analysis of surveillance data in Ireland reported that, acknowledging the number of community outbreaks reported in Ireland, it is likely that indigenous cases form the majority of illness and that among these ingestion of water and person-to-person spread are the most important mechanisms of transmission [8]. In the United States, a study evaluated sporadic cryptosporidiosis among immunocompetent persons using a case-control design. Risk factors associated with increased odds of being a case were international travel, contact with cattle, and contact with a child with diarrhoea [13]. In 2001-02, a case-control study conducted in the North West of England examined species-specific risk factors for sporadic cryptosporidiosis [9]. The authors compared risk factors for infection with genotypes 1 and 2 (currently recognised as *C. hominis and C. parvum* respectively) and found that contact with another person with diarrhoea was a risk factor for infection with *Cryptosporidium*, and that exposure through changing children's nappies was a specific risk factor for infection with *C. hominis* whether the child was symptomatic or not.

A Norwegian study looking at follow-on spread after two outbreaks where children were exposed to *Cryptosporidium* infection on a farm, found both asymptomatic and symptomatic infections in the households, which were likely to have been a result of secondary transmission [14]. Newman *et al* undertook a prospective cohort study in Brazil to examine the transmission of *Cryptosporidium* infection in households where there was an identified case [15]. Secondary cases of infection occurred in 58% of households, and around a quarter of the identified secondary cases had diarrhoea, indicating the presence of asymptomatic infection in almost three-quarters of the participants. The burden of asymptomatic infection is less well documented in *Cryptosporidium* research than for other infections but may be an important factor in household spread. A study in the UK reported a point prevalence of 1.3% among asymptomatic pre-school children attending day-care [16] suggesting that carriage does occur.

For peer review only

If person to person spread is driven by both cases and asymptomatic carriers of *Cryptosporidium*, then sporadic cases may subsequently arise following exposure to either, and outbreaks in close settings such as the home may happen more frequently than is currently recognised. Few studies exist which refute or confirm this, especially in industrialised countries.

AIMS AND OBJECTIVES

The aim of this study is to estimate the amount of secondary spread that happens in the home and describe associated factors and case characteristics. This study will support our understanding of continued apparent sporadic cryptosporidiosis in England and Wales and has implications for appropriate public health messages to help mitigate spread and infection. Further molecular characterisation of *Cryptosporidium* isolates may also help define the likelihood of secondary transmission by infecting species.

Objectives

- To estimate the number of secondary cases in households with an index case.
- To calculate the secondary transmission rate in households.
- To estimate the prevalence of asymptomatic carriage in households with an index case.
- To identify specific household-level and personal characteristics associated with secondary spread.
- To determine if factors and characteristics associated with secondary spread vary by species of *Cryptosporidium.*

METHODS

Study population

The study population will comprise residents of North West England and Wales.

For peer review only

The North West of England has a population of over 7 million people and is the third-most populated region in the United Kingdom [17]. In 2016 over 600 laboratory-confirmed *Cryptosporidium* isolates were reported from the North West (8.4/100,000 population) [18]. Wales has a total population of over 3 million people [19]. In 2016, over 400 laboratory-confirmed *Cryptosporidium* isolates were reported from Wales, the highest rate of *Cryptosporidium spp* laboratory reports per 100,000 population in England and Wales (15/100,000) [18].

Surveillance/sampling frame

The sampling frame will be taken from the two relevant surveillance systems which capture laboratory confirmed reports of *Cryptosporidium:* The Second Generation Surveillance System (SGSS) in Public Heath England (PHE), and Tarian in Public Health Wales (PHW). Systematic national surveillance of laboratory confirmed *Cryptosporidium* in England and Wales has been established for many years [20]. Positive samples identified in the diagnostic laboratories are also routinely forwarded to the national *Cryptosporidium* Reference Unit (CRU) which provides expert management, prevention and control advice as well as *Cryptosporidium* typing and confirmation services for speciation and surveillance [21].

All cases of laboratory confirmed *Cryptosporidium sp*. reported from primary diagnostic microbiology laboratories in North West England and Wales, in the study year, will initially be eligible.

Study type

The identification of cases, and their subsequent recruitment, is cross-sectional, although the study also involves retrospective data collection and some prospective sampling (**Error! Reference source not found.**).

Index cases of cryptosporidiosis will be identified via the relevant surveillance system(s).

Once participants are recruited they will complete a questionnaire (one per household),

For peer review only

collecting clinical, demographic, and household composition information. In addition, all household members (excluding the index case) will be asked to supply a stool sample.

Study period

The study period will be 12 months, to account for seasonal variation and allow maximum enrolment, up to 400 households. The study is expected to begin with a pilot phase of 1-2 months in autumn 2018.

Sample size

Given that the North West & Wales report around 1,000 cases per year (PHE data, 2015) and assuming a participation rate of 40-60%, and some exclusions (based on similar studies/approaches [11,22]) we anticipate a sample size of 400 households. Using 2011 Census indications of 2.4 persons on average per household[17], we can expect to recruit around 960 -1,000 participants in total. Using the outcome of at least one further secondary case within 0 - 20% of households [11,14,15,23–25], a range of sample sizes can be estimated. However, recruitment of 400 households is feasible, and sufficient to allow us to demonstrate a statistically significant minimum odds/risk ratio of 2.0, with type 1 error 0.05 and type 2 error at 0.20.

For peer review only

234

235 **Case definition(s)**

236 Tables 1 and 2 outline case and household definitions used to categorise household members and define secondary transmission.

Case definitions
**Index case**<br><br>The first case from a household identified in the surveillance system (person reported to a PHE/PHW surveillance system(s) following detection of *Cryptosporidium sp.* in a faecal sample, with a specimen date in the study year)<br><br>**Secondary case(s)**<br>Probable secondary case<br>A person in a household of an index case, with symptoms:<br>of diarrhoea and/or vomiting<br>AND<br>that started after another case's onset date in the household<br>Confirmed secondary case<br>A person in a household of an index case, with symptoms:<br>of diarrhoea and/or vomiting<br>AND<br>That started after another case's onset date in the household;<br>AND<br>a *Cryptosporidium* positive stool sample<br>**Asymptomatic**<br>A person in a household of an index case with:<br>no reports of gastrointestinal illness;<br>AND<br>a *Cryptosporidium* positive stool sample.

237 *Table 1: Case definitions*

For peer review only

Household definitions
**Household**
Two or more people (not necessarily related) living at the same address in North West England or Wales who share cooking facilities and share a living room or sitting room or dining area [18].
**Household member**
A person who normally resides in the household and regularly shares food or toilet facilities [26]
**Household contact**
A household member where an index case has been identified
**Household with transmission**
A household that has more than one case
**Household without transmission**
A household that has one case (the index case)

238 *Table 2: Household definitio*

For peer review only

Recruitment approach

Overview and rationale

Cases of cryptosporidiosis are identified from routine surveillance (from diagnostic laboratories) and are contacted via post, by the relevant public health organisation, in the first instance. Following this, if they do not opt-out, they are contacted via telephone by an NHS research nurse at the local Clinical Research Network (CRN) to chat about the study and determine if they would like to take part.

Our approach to the recruitment process was driven by necessity and feasibility and we explored several options at the protocol drafting stage of the project, balancing data needs with patient choice. As detection of *Cryptosporidium* is retrospective and undertaken by laboratory staff, there is no opportunity to consent individuals at the time of diagnosis and the recruitment process could not be achieved without access to patient information. In our model, participants are given opt-out options at each contact and it is emphasised that they can withdraw at any time. Previous research supports the acceptability and understanding of this method, recognising that an approach of 'consent for each use' is burdensome for both researcher and participant [27] [28], as does patient response and engagement with similar studies.[1]

Public and Patient Involvement

Patients and public were not involved in the overall design of the study, but we did elicit some public opinion when finalising our approach to recruitment. Following valuable comments from the ethical review board we undertook a short survey among the public and specific Patient and Public Involvement (PPI) groups to gauge general attitudes toward accessing data prior to consent, to support recruitment to research. We drafted a survey which outlined the approach to recruitment and the framework of the study. We accessed a lay PPI group from the Infection and Global Heath panel at the University of Liverpool, and

[1] Studies recruiting based on disease surveillance are common for GI infections, and many projects have taken this approach – the methodology for the epiCrypt Study has been influenced by design aspects of large-scale studies such as Enigma, IID2, and Integrate.

For peer review only - http://bmjopen.bmj.com/site/about/guidelines.xhtml

one from Health and Care Research Wales. Participants were asked to think generally about the method of recruitment and how they felt about this approach. In general, the feeling was that it is acceptable to access data for recruitment, especially to support much needed research. However, considerations and worries included the person accessing data, with NHS/public health staff generally viewed as more favourable that non-NHS (e.g. University) researchers.

Identification and first contact with the index case

Laboratory diagnosed reports of *Cryptosporidium*, and the corresponding patient contact details, will be extracted from the relevant surveillance system by health protection staff and saved in a line list.

All potentially eligible participants will be issued a unique sequential study ID by PHE/PHW staff in the initial line listing. This will be on all relevant study documentation and stool pots and follow each person and household through the study journey. This will allow data to be linked pseudonymously and helps with data management.

Staff from either PHE or PHW (depending on case location) will send an invite letter through the post to these potentially eligible index cases. The invite letter outlines the study, describes why the case has been contacted, and explains that a research nurse may be in touch over the coming weeks to discuss the study. The letter allows the case to choose several ways of opting-out of this contact (e-mail, freepost, telephone) and provides a named, clinical study lead for each public health organisation should they wish to discuss any aspect of this.

Approaching to recruit

If a contacted case does not opt-out within two weeks, their details will be shared securely (using internally agreed practices) with the NHS research nurses at the Clinical Research Network North West Coast (CRN). The research nurses will attempt to contact the index case (or parent/guardian of,) via telephone (using internally agreed practices) to inform them about the study and offer them the opportunity to participate, if eligible. A maximum of three

For peer review only

attempts will be made, and nurses will not leave voicemails. If a case is unable to be contacted, or does not wish to participate at this stage, their details will be deleted from the line list. If the approached index case is successfully contacted via telephone and interested in participating, or would like more information, the research nurses will prepare and post a study pack. Index cases may be excluded at this stage where discussions with the case reveal that any of the following exclusion criteria apply;

- Index case is in a single-person household
- Index case is a visitor to a household in the study area, but is registered with a GP outside the study area
- Household is outside the study area
- The index case is resident in an institution: retirement home, nursing home, prison, barracks, boarding school, or college/university halls of residence

Study packs

The study packs contain:

- A study information pamphlet
- A questionnaire booklet for the index case or a suitable representative (e.g. parent, head of household) to complete, with a freepost envelope
- A consent form for each participating household member to read, initial, and sign (forms part of the questionnaire)
- A stool sampling pack (Fe-Col®) for each participating household member, with the required return postal envelope
- An information leaflet on cryptosporidiosis and the relevant health advice
- An information sheet on General Data Protection Regulation (GDPR) for health and care research

Consent

For peer review only

The index case and any household members who wish to take part will sign and return the consent form at the front of the questionnaire. The return of study materials such as a completed questionnaire and/or stool samples will be considered implied consent.

Disenrollment

If study materials are not received within 14 days of posting the pack, a reminder letter will be dispatched by the research nurses at the CRN. If study documentation is not returned within 14 days of posting the reminder letter, no further attempt at contact will be made and the index case will be removed from the study line list.

Participation

If the household wants to take part all interested members will sign and return the consent form. At least one household member, as well as the index case, must consent.

Consent forms and questionnaires are returned to the University of Liverpool in a stamped addressed envelope provided in the pack. The unique study IDs of those consenting will be shared weekly with the research nurses at the CRN to cross-match those contacted index cases that have been recruited and enrolled. Each consenting household member will also be asked to provide a stool sample for testing, using the provided Fe-Col® kits, which include a pre-addressed and secure postal bag (compliant with UN3373 regulations for mailing Cat B biological samples [29]). Instructions are provided, and samples will be returned directly to the *Cryptosporidium* Reference Unit.

For peer review only

Data management and oversight

A flow chart outlining the data management and responsibilities of each organisation is shown in Figure 2.

Documentation

Questionnaire data will be inputted from the paper format to a corresponding MS Access database and held securely on a University of Liverpool drive in accordance with their security protocols. Double data entry will be undertaken on a sample of questionnaires and

discrepancies resolved using internal validation checks. When data entry is complete the data will be exported to the final study database [MS Access] where the data are pseudonymised for analyses. Name, date of birth, and full postcode will be removed and replaced with unique study ID, age, and Lower Super Output Area (LSOA).

All data storage, cleaning and analyses will be undertaken by the study team at the University of Liverpool. Data will be stored on institutional network drives with appropriate security measures in place. Hard copy records will be stored in a locked cabinet in a secure location and access to records and data are limited to study personnel. Paper documents will be stored separately from the corresponding electronic data. The sponsor and data controller for this study is the Clinical Governance Team at the University of Liverpool.

Data security protocol available on request

Stool sample management

For purposes of data confidentiality and governance, stool samples returned to the *Cryptosporidium* Reference Unit are pseudonymised with the unique study ID, and participants will be asked to write their age, sex, and date of sample on their sample pot before they collect the stool. Stool sample results will be added to a study-specific database held at the CRU. The stool sample results will be added to the study database at the end of data collection using unique study ID in a secure file transfer.

Identifying the index case samples

Original diagnostic laboratory numbers will be retained with the index case information in the original line lists at PHE/PHW so that the sample can be identified later at the CRU and grouped with the relevant household samples. This sample, when located at the CRU, will be processed in the same way as other study samples.

Full laboratory protocol available on request

For peer review only

Outcomes and measurements

Questionnaire data

The questionnaire is divided into sections and is mostly composed of dichotomous and multiple-choice questions. Section A asks questions to determine the composition of the household, the clinical details of the index case, and captures any other symptomatic household members. A table is used to collect information on any other symptomatic diarrhoeal illness in the house and will attempt to capture relationships to the index case (Table 3).

Age	*Sex*	*Relationship to first case*	*Been ill with diarrhoea and/or vomiting*<br><br>*[Yes/No/Don't know]*	*When they became ill*<br><br>*[Date if known, otherwise before/after the first case]*	*How many days were they ill with these symptoms?*	*Did they see a doctor about this illness?*
39	*F*	*Mother*	*Yes*	*18/12/2017*	*10*	*No*
42	*M*	*Father*	*Yes*	*Before*	*About 3 days*	*No*
6 months	*M*	*Brother*	*No*	*-*		
24	*F*	*Lodger/House mate*	*Don't know*	*-*		

Table 3: An example table showing information collected in the questionnaire about other illness in the house

Section B records activities of the index case, and others in the home, in the two weeks prior to the index case's onset, based on known exposures for *Cryptosporidium*. Information on outdoor and leisure activities may help determine possible co-primary infections and distinguish them from those that are secondary. (Table 4)

Sections C and D collect household variables, including the number of bedrooms and bathrooms, and capturing those who share beds/baths, and asking about outside space and

For peer review only

383 animals. We also ask about nappy changing and toilet training in the home, and about
384 general hand-washing behaviour.

385 *Full questionnaire available on request.*

386

387

388

389

Activity	FIRST CASE	ANYONE ELSE IN THE HOME
Travel outside the UK		
Swimming – outdoors in a lake, river, stream etc. (wild swimming)		
Swimming – in a treated swimming pool, either indoors or outdoors (such as a pool at a leisure centre or a lido)		
Other water activities/sport (such as surfing, rowing, water-skiing etc.)		
Other outdoor activities (such as camping, climbing, hiking, cycling etc.)		
Gardening (at home or elsewhere, such as an allotment)		
Contact with pets (at home or with pets at another house)		
Visiting or working on a farm or had contact with farm animals		
Visiting or working at a zoo or had contact with zoo or wild animals		

390 *Table 4: An example table showing information collected in the questionnaire about activities*

391

392

393

For peer review only

Stool collection and genotyping

All household members of the index case (but not the index case) will be asked to provide a stool sample using the Fe-Col® kit provided in the study pack, and post to the *Cryptosporidium* Reference Unit. (Figure 3)

The stool pots will be labelled with the unique household ID which identifies them as part of the study but allows the samples to remain anonymous to the reference laboratory team. Samples will be scored against the Bristol stool scale and tested, only for *Cryptosporidium*, using Immunofluorescence (IF) microscopy. Positive samples will be further processed by oocyst purification and DNA extraction and then *Cryptosporidium* speciated using validated PCR techniques which are part of normal practice [30]. *Cryptosporidium* DNA will be retained for subtyping and possibly whole genome sequencing (WGS) at a later date.

Full laboratory protocol available on request

ANALYSES

The primary objective of this study is to determine the amount of secondary spread that happens in the home following exposure to a case. This will be established by testing stool samples of household members of a case for *Cryptosporidium* and reporting the numbers of secondary cases (according to our pre-determined definitions). A household with more than one case will be a household with secondary transmission.

We will calculate the following:

- The secondary transmission rate/prevalence within households (number of cases in the home/numbers at risk in the home, number of households with secondary spread /number of households)
- The amount of asymptomatic carriage among those exposed to symptomatic cases (number of asymptomatic cases/number at risk)
- Odds/Risk (OR/RR) of secondary illness according to activities and case/household characteristics

For peer review only

- Odds/Risk (OR/RR) of secondary illness according to organism species

Confounding (e.g. host factors such as age, co-morbidity) will be considered, where known, using multivariable techniques. Also, we will, where possible, examine environmental level exposures using stratification, e.g. those households/cases which are exposed to other known sources or risk factors, such as those living on farms.

Data will be analysed, where appropriate, using Stata v12.

For peer review only

LIMITATIONS AND BIASES

Some elements of the study design are retrospective in nature, as the index case must have already been ill and been tested in order to be selected. As a result, some ascertainment bias may lead to a skewed sample from which to choose the index cases. We do not expect to capture the full profile of cases and households in the population that might have *Cryptosporidium* due to differences in risk or vulnerability, severity, or health-seeking behaviour[31]. As *Cryptosporidium* is common in younger age groups we expect a large proportion of the participants to represent families with young children. Any likely over- or under-representation in the data collected will be considered when assessing and describing results. Further unidentifiable limitations may include recall biases around dates of onset or activities, and classification biases as we are asking about self-reported illness and information may be inaccurate.

There is a possibility that we could see ongoing outbreaks in the study year. If this happens, we might try to identify these where possible and may consider excluding households from this study where all or most members have been exposed, including a definition of aco-primary case.

End of study

The study will be declared as ended when the database is closed to recruitment – after one year or when the maximum number of households has been enrolled.

Pilot arrangements

A pilot phase of one month is anticipated before data collection begins to assess and evaluate processes. Pilot data will be included as study data if no major methodological changes are proposed.

Ethics and dissemination

The study has been approved by the North West – Liverpool East NHS Research Ethics Committee (REC Reference: 18/NW/0300) and the Confidentiality and Advisory Group (Reference 18/CAG/0084). The project is registered on the National Institute for Health Research (NIHR) portfolio (CPMS ID – 39458).

Outputs will include scientific conferences and peer-reviewed publications. In addition, a short, lay report of findings will be produced for participants, who can opt to receive this when they take part.

References

1 Chalmers RM, Smith R, Elwin K, *et al.* Epidemiology of anthroponotic and zoonotic human cryptosporidiosis in England and Wales, 2004 to 2006. *Epidemiol Infect* 2011;**139**:700–12.http://www.jstor.org/stable/27975646

2 Cacciò SM, Putignani L. Epidemiology of Human Cryptosporidiosis. In: *Cryptosporidium: parasite and disease*. Vienna: : Springer Vienna 2014. 43–79. doi:10.1007/978-3-7091-1562-6_2

For peer review only

3 Adak GK, Long SM, O'Brien SJ. Trends in indigenous foodborne disease and deaths, England and Wales: 1992 to 2000. *Gut* 2002;**51**:832–41.http://www.ncbi.nlm.nih.gov/pubmed/12427786 (accessed 12 Jun 2017).

4 Food Standards Agency. A report of infectious intestinal disease in England. London: 2000.

5 Cacciò SM, Thompson RCA, McLauchlin J, *et al.* Unravelling Cryptosporidium and Giardia epidemiology. *Trends Parasitol* 2005;**21**:430–7. doi:10.1016/j.pt.2005.06.013

6 Chalmers RM, Giles M. Zoonotic cryptosporidiosis in the UK – challenges for control. *J Appl Microbiol* 2010;**109**:1487–97. doi:10.1111/j.1365-2672.2010.04764.x

7 Hannah J, Riordan T. Case to case spread of cryptosporidiosis; evidence from a day nursery outbreak. *Public Health* 1988;**102**:539–44.https://www.ncbi.nlm.nih.gov/pubmed/3231695

8 Garvey P, McKeown P. EPIDEMIOLOGY OF HUMAN CRYPTOSPORIDIOSIS IN IRELAND, 2004-2006: ANALYSIS OF NATIONAL NOTIFICATION DATA. *EUROSURVEILLANCE* 2009;**14**:16–20.

9 Hunter PR, Hughes S, Woodhouse S, *et al.* Sporadic cryptosporidiosis case-control study with genotyping. *Emerg Infect Dis* 2004;**10**:1241–9. doi:10.3201/eid1007.030582

10 Nichols G, Chalmers R, Lake I, *et al.* Cryptosporidiosis: A report on the surveillance and epidemiology of Cryptosporidium infection in England and Wales . 2006. doi:08/11/2006

11 Waldram A, Vivancos R, Hartley C, *et al.* Prevalence of Giardia infection in households of Giardia cases and risk factors for household transmission. *BMC Infect Dis* 2017;**17**:486. doi:10.1186/s12879-017-2586-3

12 Minetti C, Lamden K, Durband C, *et al.* Case-Control Study of Risk Factors for Sporadic Giardiasis and Parasite Assemblages in North West England. *J Clin Microbiol* 2015;**53**:3133–40. doi:10.1128/JCM.00715-15

13 Abubakar I, Aliyu S, Arumugam C, *et al.* Risk factors for sporadic cryptosporidiosis

For peer review only

among immunocompetent persons in the United States from 1999 to 2001. *J Clin Microbiol* 2004;**37**:2944–51. doi:10.3201/eid0905.020678

14 Johansen OH, Hanevik K, Thrana F, *et al.* Symptomatic and asymptomatic secondary transmission of Cryptosporidium parvum following two related outbreaks in schoolchildren. *Epidemiol Infect* 2014;**143**:1–8. doi:10.1017/S095026881400243X

15 Newman RD, Zu SX, Wuhib T, *et al.* Household epidemiology of Cryptosporidium parvum infection in an urban community in Northeast Brazil. *Ann Intern Med* 1994;**120**:500–5.http://www.ncbi.nlm.nih.gov/pubmed/8311373 (accessed 27 Apr 2017).

16 Davies A, Campbell B, Evans MR, *et al.* Asymptomatic carriage of protozoan parasites in children in day care centers in the United Kingdom. *Pediatr Infect Dis J* 2009;**28**:838–40.https://insights.ovid.com/pubmed?pmid=19684527

17 ONS. 2011 Census - Office for National Statistics. https://www.ons.gov.uk/peoplepopulationandcommunity/populationandmigration/populationestimates/bulletins/populationandhouseholdestimatesfortheunitedkingdom/2011-03-21#number-of-people-in-households (accessed 11 Jun 2018).

18 PHE. Cryptosporidium: national laboratory data - GOV.UK. https://www.gov.uk/government/publications/cryptosporidium-national-laboratory-data (accessed 11 Jun 2018).

19 Welsh Government | Census of population. Stat. Res. 2015.https://gov.wales/statistics-and-research/census-population/?lang=en (accessed 10 Aug 2018).

20 Wall PG, de Louvois J, Gilbert RJ, *et al.* Food poisoning: notifications, laboratory reports, and outbreaks--where do the statistics come from and what do they mean? *Commun Dis Rep CDR Rev* 1996;**6**:R93-100.http://www.ncbi.nlm.nih.gov/pubmed/8680502 (accessed 10 Aug 2018).

21 Cryptosporidium reference unit (CRU) - GOV.UK. https://www.gov.uk/guidance/cryptosporidium-reference-unit-cru (accessed 10 Aug

For peer review only

2018).

22 Tam CC, Rodrigues LC, Viviani L, *et al.* Longitudinal study of infectious intestinal disease in the UK (IID2 study): incidence in the community and presenting to general practice. *Gut* 2012;**61**:69–77. doi:10.1136/gut.2011.238386

23 Perry S, De La Luz Sanchez M, Hurst PK, *et al.* Household transmission of gastroenteritis. *Emerg Infect Dis* 2005;**11**:1093–6. doi:10.3201/eid1107.040889

24 Harrison SL, Nelder R, Hayek L, *et al.* Managing a large outbreak of cryptosporidiosis: how to investigate and when to decide to lift a 'boil water' notice. *Commun Dis public Heal* 2002;**5**:230–9.

25 MacKenzie WR, Schell WL, Blair KA, *et al.* Massive outbreak of waterborne cryptosporidium infection in Milwaukee, Wisconsin: recurrence of illness and risk of secondary transmission. *Clin Infect Dis* 1995;**21**:57–62.http://www.ncbi.nlm.nih.gov/pubmed/7578760 (accessed 27 Apr 2017).

26 Public health control and management of hepatitis A 2017 Guidelines. Published Online First: 2017.https://www.gov.uk/government/uploads/system/uploads/attachment_data/file/623036/Public_health_control_and_management_of_hepatitis_A_2017.pdf (accessed 30 Oct 2017).

27 Willison DJ, Swinton M, Schwartz L, *et al.* Alternatives to project-specific consent for access to personal information for health research: insights from a public dialogue. *BMC Med Ethics* 2008;**9**:18. doi:10.1186/1472-6939-9-18

28 Taylor MJ, Taylor N. Health research access to personal confidential data in England and Wales: assessing any gap in public attitude between preferable and acceptable models of consent. *Life Sci Soc policy* 2014;**10**:15. doi:10.1186/s40504-014-0015-6

29 Category B - Medical Packaging, medical pouches and biological substances on un3373.com. http://www.un3373.com/category-biological-substances/category-b/ (accessed 10 Aug 2018).

30 Robinson G EK and CR. Methods and Protocols in Cryptosporidium Research.

For peer review only

Cryptosporidium diagnostic assays: molecular detection. In: Ed. Mead J, ed. *Methods in Molecular Biology*. Springer Nature

31 Adams NL, Rose TC, Hawker J, *et al.* Socioeconomic status and infectious intestinal disease in the community: a longitudinal study (IID2 study). *Eur J Public Health* 2018;**28**:134–8. doi:10.1093/eurpub/ckx091

Authors' contributions

CMCK, RCha, RV, RChr, SOB conceived the initial idea for the study and the methodology. JM and the CRN team contributed to methods for participant recruitment and ethics submissions. CMCK wrote the protocol. RChr, RCha, RV, SOB reviewed and revised the protocol and the paper. All authors have read and approved the final manuscript.

Funding statement

This research is jointly funded by the National Institute for Health Research Health Protection Research Unit (NIHR HPRU) in Emerging and Zoonotic Infections (HPRU EZI) and the National Institute for Health Research Health Protection Research Unit (NIHR HPRU) in Gastrointestinal Infections (HPRU GI) at the University of Liverpool in partnership with Public Health England (PHE), University of East Anglia, University of Oxford and the Institute of Food Research. The views expressed are those of the authors and not necessarily those of the NHS, the NIHR, the Department of Health, PHE or Public Health Wales.

Competing interests statement

The authors declare that they have no competing interests.

Acknowledgements

John Harris, Goutam Adak, Ken Lamden, Andrew Fox

The Clinical Research Network North West Coast, Liverpool

For peer review only

581 Laura Evans, Public Health Wales

582 Christopher Williams and the Health Protection Team, Public Health Wales

583 Jo Hardstaff, University of Liverpool

584 Richard Dunn and Elaine Mooney, Field Services, Public Health England

585 Infection and Global Heath panel (PPI group) at the University of Liverpool, Health and Care
586 Research Wales, and all other public/lay contributors to the Public and Patient Involvement
587 survey.

588

589 **Figure Legends**

590 Figure 1: Outline of study design and main objectives

591 Figure 2: Study flow diagram outlining the flow of recruitment and the roles of each
592 organisation

593 Figure 3: Fe-Col® kit for stool collection

594

595

For peer review only

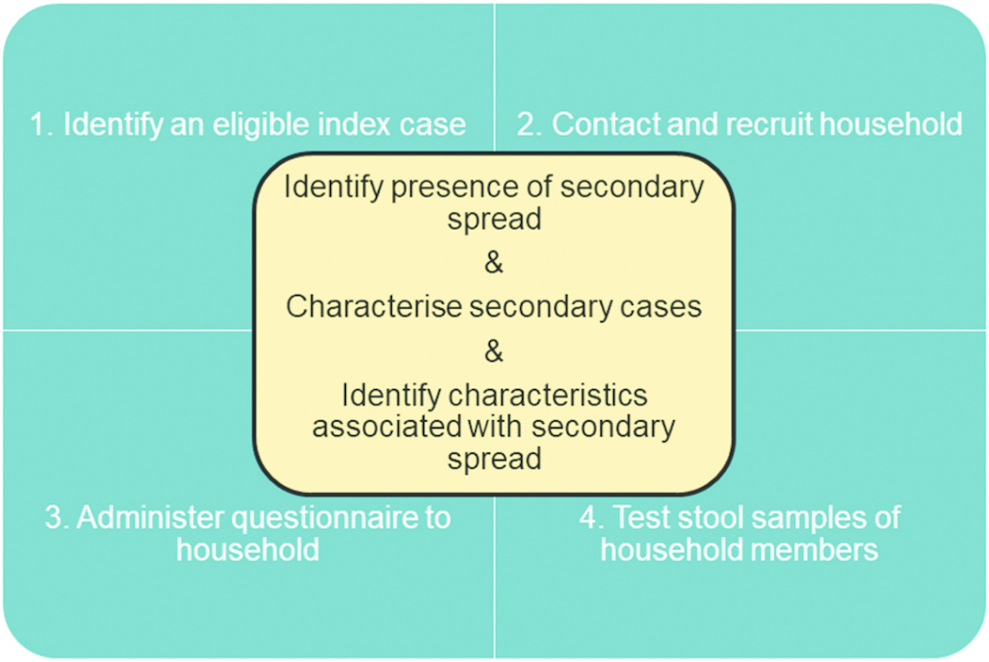

Outline of study

90x90mm (300 x 300 DPI)

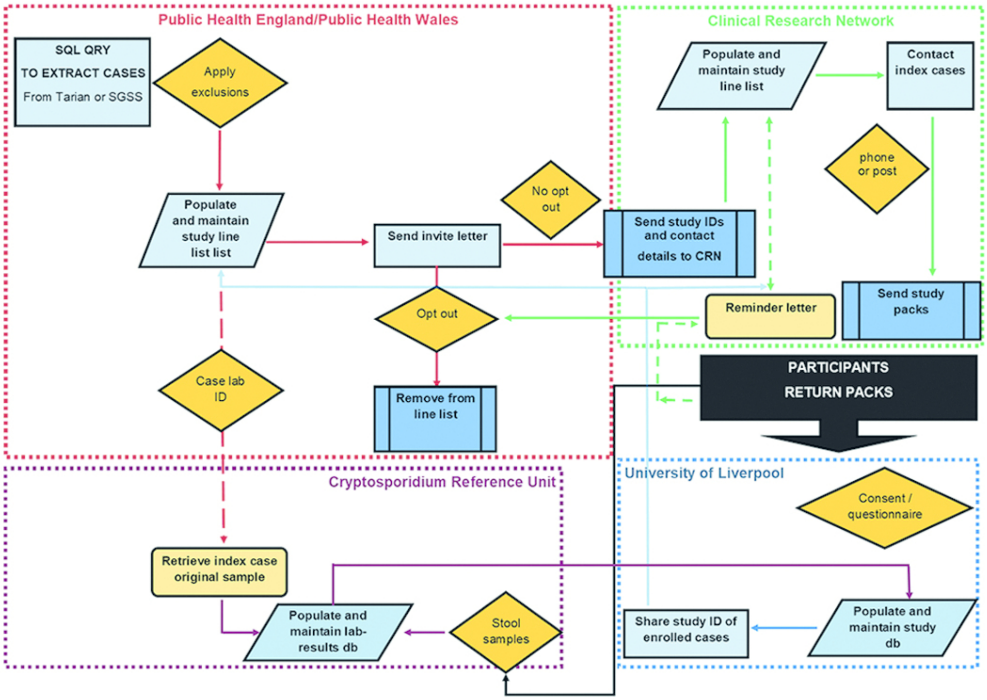

Study Flow Diagram

90x90mm (300 x 300 DPI)

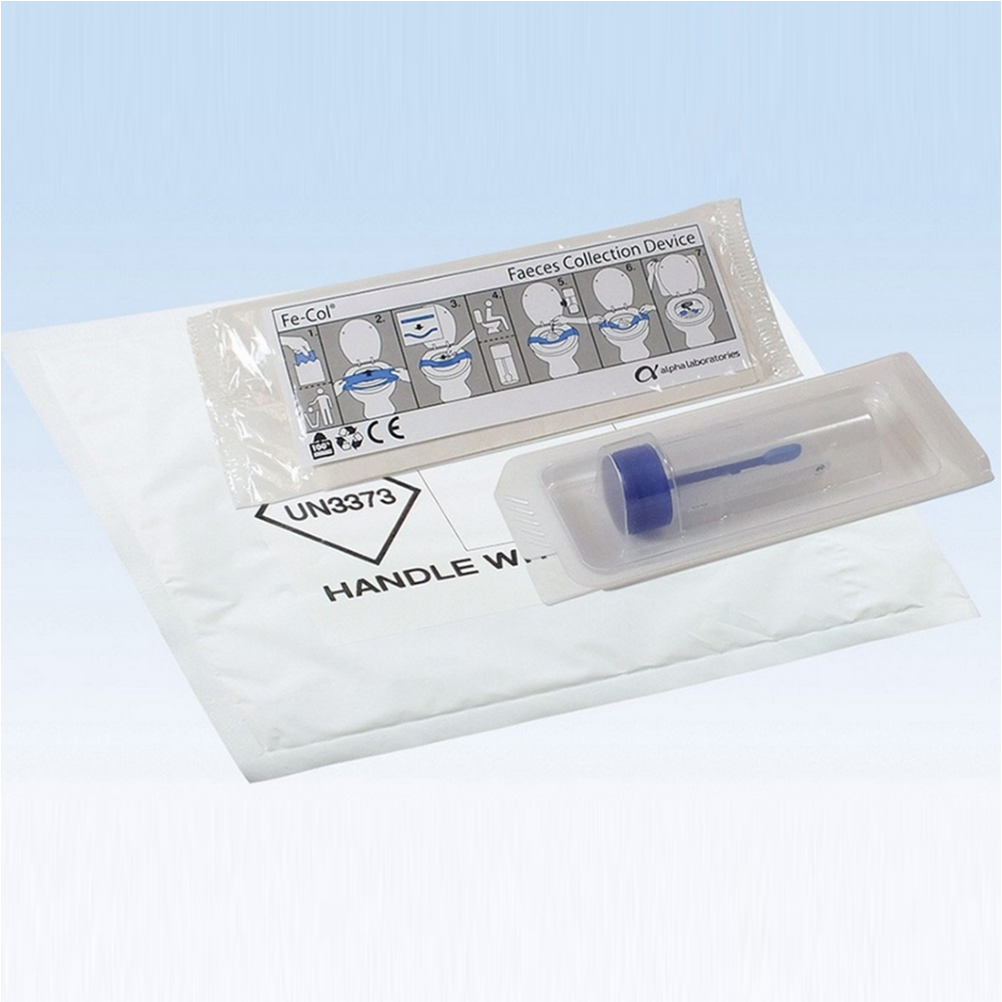

Fe-Col kit

90x90mm (300 x 300 DPI)

STROBE Statement—checklist of items that should be included in reports of observational studies

	Item No	Recommendation
**Title and abstract**	1	(*a*) Indicate the study's design with a commonly used term in the title or the abstract
		(*b*) Provide in the abstract an informative and balanced summary of what was done and what was found
**Introduction**		
Background/rationale	2	Explain the scientific background and rationale for the investigation being reported
Objectives	3	State specific objectives, including any prespecified hypotheses
**Methods**		
Study design	4	Present key elements of study design early in the paper
Setting	5	Describe the setting, locations, and relevant dates, including periods of recruitment, exposure, follow-up, and data collection
Participants	6	(*a*) *Cohort study*—Give the eligibility criteria, and the sources and methods of selection of participants. Describe methods of follow-up<br>*Case-control study*—Give the eligibility criteria, and the sources and methods of case ascertainment and control selection. Give the rationale for the choice of cases and controls<br>*Cross-sectional study*—Give the eligibility criteria, and the sources and methods of selection of participants
		(*b*) *Cohort study*—For matched studies, give matching criteria and number of exposed and unexposed<br>*Case-control study*—For matched studies, give matching criteria and the number of controls per case
Variables	7	Clearly define all outcomes, exposures, predictors, potential confounders, and effect modifiers. Give diagnostic criteria, if applicable
Data sources/ measurement	8*	For each variable of interest, give sources of data and details of methods of assessment (measurement). Describe comparability of assessment methods if there is more than one group
Bias	9	Describe any efforts to address potential sources of bias
Study size	10	Explain how the study size was arrived at
Quantitative variables	11	Explain how quantitative variables were handled in the analyses. If applicable, describe which groupings were chosen and why
Statistical methods	12	(*a*) Describe all statistical methods, including those used to control for confounding we are calculating ORs and percentages. Have outlined objectives.
		(*b*) Describe any methods used to examine subgroups and interactions
		(*c*) Explain how missing data were addressed
		(*d*) *Cohort study*—If applicable, explain how loss to follow-up was addressed<br>*Case-control study*—If applicable, explain how matching of cases and controls was addressed<br>*Cross-sectional study*—If applicable, describe analytical methods taking account of sampling strategy
		(*e*) Describe any sensitivity analyses

Comment [cmck1]: These may not be known until after the study data are analysed.
This is a protocol paper.
We do describe that we will use stratification for sub-groups if data are relevant.

Continued on next page

For peer review only - http://bmjopen.bmj.com/site/about/guidelines.xhtml

**Results n/a**		
Participants	13*	(a) Report numbers of individuals at each stage of study—eg numbers potentially eligible, examined for eligibility, confirmed eligible, included in the study, completing follow-up, and analysed
		(b) Give reasons for non-participation at each stage
		(c) Consider use of a flow diagram
Descriptive data	14*	(a) Give characteristics of study participants (eg demographic, clinical, social) and information on exposures and potential confounders
		(b) Indicate number of participants with missing data for each variable of interest
		(c) *Cohort study*—Summarise follow-up time (eg, average and total amount)
Outcome data	15*	*Cohort study*—Report numbers of outcome events or summary measures over time
		Case-control study—Report numbers in each exposure category, or summary measures of exposure
		Cross-sectional study—Report numbers of outcome events or summary measures
Main results	16	(*a*) Give unadjusted estimates and, if applicable, confounder-adjusted estimates and their precision (eg, 95% confidence interval). Make clear which confounders were adjusted for and why they were included
		(*b*) Report category boundaries when continuous variables were categorized
		(*c*) If relevant, consider translating estimates of relative risk into absolute risk for a meaningful time period
Other analyses	17	Report other analyses done—eg analyses of subgroups and interactions, and sensitivity analyses
**Discussion**		
Key results	18	Summarise key results with reference to study objectives
Limitations	19	Discuss limitations of the study, taking into account sources of potential bias or imprecision. Discuss both direction and magnitude of any potential bias
Interpretation	20	Give a cautious overall interpretation of results considering objectives, limitations, multiplicity of analyses, results from similar studies, and other relevant evidence
Generalisability	21	Discuss the generalisability (external validity) of the study results
**Other information**		
Funding	22	Give the source of funding and the role of the funders for the present study and, if applicable, for the original study on which the present article is based

Comment [cmck2]: N/A Protocol

*Give information separately for cases and controls in case-control studies and, if applicable, for exposed and unexposed groups in cohort and cross-sectional studies.

Note: An Explanation and Elaboration article discusses each checklist item and gives methodological background and published examples of transparent reporting. The STROBE checklist is best used in conjunction with this article (freely available on the Web sites of PLoS Medicine at http://www.plosmedicine.org/, Annals of Internal Medicine at http://www.annals.org/, and Epidemiology at http://www.epidem.com/). Information on the STROBE Initiative is available at www.strobe-statement.org.

For peer review only

The epiCrypt study protocol: a cross-sectional investigation of household transmission of Cryptosporidium in England and Wales

Journal:	*BMJ Open*
Manuscript ID	bmjopen-2018-026116.R1
Article Type:	Protocol
Date Submitted by the Author:	14-Feb-2019
Complete List of Authors:	McKerr, Caoimhe; university of liverpool, NIHR Health Protection Research Unit in Gastrointestinal Infections; University of Liverpool, NIHR Health Protection Research Unit in Emerging and Zoonotic Infections<br>Chalmers, Rachel; Public Health Wales, Cryptosporidium Reference Unit; Swansea University Medical School<br>Vivancos, Roberto; Public Health England, Field Epidemiology Services, Health Protection; The University of Liverpool, NIHR Health Protection Research Unit in Emerging and Zoonotic Infections<br>O'Brien, Sarah; University of Liverpool, Institute of Psychology, Health and Society; University of Liverpool, NIHR Health Protection Research Unit in Gastrointestinal Infections<br>Mugarza, Julie; NIHR Clinical Research Network North West Coast<br>Christley, Robert; University of Liverpool, Department of Epidemiology and Population Health, Institute of Infection and Global Health; NIHR Health Protection Research Unit in Emerging and Zoonotic Infections
**Primary Subject Heading**:	Epidemiology
Secondary Subject Heading:	Epidemiology, Infectious diseases
Keywords:	Cryptosporidium, Household transmission, Secondary spread, Zoonoses, Gastrointestinal infection, Epidemiology < TROPICAL MEDICINE

Short title

The epiCrypt Study - protocol

Word Count

≈4100 (excl. figs, tbls, refs)

Authors:

Caoimhe McKerr[1,2,6] / c.mckerr@liv.ac.uk

Rachel M Chalmers[3,7] / rachel.chalmers@wales.nhs.uk

Roberto Vivancos[1,2,4] / roberto.vivancos@phe.gov.uk

Sarah J O'Brien[1] / sjobrien@liv.ac.uk

Julie Mugarza[5] / julie.mugarza@nihr.ac.uk

Robert M Christley[2,6] / robc@liv.ac.uk

1 NIHR Health Protection Research Unit in Gastrointestinal Infections, The University of Liverpool

2 NIHR Health Protection Research Unit in Emerging and Zoonotic Infections, The University of Liverpool

3 *Cryptosporidium* Reference Unit, Public Health Wales, Swansea

4 Field Epidemiology Services, Public Health England, Liverpool

5 NIHR Clinical Research Network (CRN) North West Coast, Liverpool

6 Institute of Infection and Global Health, University of Liverpool

7 Swansea Medical School, Swansea University

For peer review only

Corresponding author:

Caoimhe McKerr

Current address:

The University of Liverpool

Institute of Infection and Global Health

Waterhouse Building (2nd Floor, Block F)

1-5 Brownlow Street

Liverpool L69 3GL

Email: c.mckerr@liverpool.ac.uk

For peer review only

TITLE

The epiCrypt study protocol: a cross-sectional investigation of household transmission of *Cryptosporidium* in England and Wales

AUTHORS

Caoimhe McKerr[1,2,6] / c.mckerr@liv.ac.uk

Rachel M Chalmers[3,7] / rachel.chalmers@wales.nhs.uk

Roberto Vivancos[1,2,4] / roberto.vivancos@phe.gov.uk

Sarah J O'Brien[1] / sjobrien@liv.ac.uk

Julie Mugarza[5] / julie.mugarza@nihr.ac.uk

Robert M Christley[2,6] / robc@liv.ac.uk

1 NIHR Health Protection Research Unit in Gastrointestinal Infections, The University of Liverpool

2 NIHR Health Protection Research Unit in Emerging and Zoonotic Infections, The University of Liverpool

3 *Cryptosporidium* Reference Unit, Public Health Wales, Swansea

4 Field Epidemiology Services, Public Health England, Liverpool

5 NIHR Clinical Research Network (CRN) North West Coast, Liverpool

6 Institute of Infection and Global Health, University of Liverpool

7 Swansea Medical School, Swansea University

Correspondence to: c.mckerr@liv.ac.uk

For peer review only

ABSTRACT

Introduction

Infection with the *Cryptosporidium* parasite causes over 4,000 cases of diagnosed illness (cryptosporidiosis) in England and Wales each year. Risk factors are often estimated from outbreak investigations, and in the UK include ingestion of contaminated water and food, farm/animal contact and person-to-person spread in institutions. However, reported outbreaks only represent about 10% of cases and the transmission routes for sporadic disease may not be the same. Contact with other people has been highlighted as a factor in the transmission of *Cryptosporidium*, but the incidence of sporadic disease has not been sufficiently established, and how frequently this arises from contact with other infected people is not well documented.

This project will estimate the amount of secondary spread that occurs in the home and potentially identify asymptomatic infections which might have a role in transmission. Risk factors and characteristics associated with secondary spread will be described including any differences in transmission between *Cryptosporidium* species.

Methods and analysis

The study will prospectively identify cryptosporidiosis cases from North West England and Wales over one year and invite them and their household to take part. Each household will complete a questionnaire and each household member will be asked to provide a stool sample. Clinical, demographic, and home variables will be described, and further analyses undertaken to investigate associations with secondary spread in the home.

Cryptosporidium-positive stool samples, identified by immunofluorescence microscopy, will be characterised using molecular methods to describe patterns of transmission. Data collection is expected to take one year, beginning in September 2018.

Ethics

The study has been approved by the North West – Liverpool East NHS Research Ethics Committee (Reference: 18/NW/0300) and the Confidentiality and Advisory Group (Reference

For peer review only

18/CAG/0084). The project is registered on the National Institute for Health Research (NIHR) portfolio (CPMS ID: 39458).

KEYWORDS

Cryptosporidium – Protozoa – Sporadic disease – Household transmission – Person-to-person – Secondary spread - Zoonoses – Gastrointestinal infection – Risk factors – Epidemiology - Parasite – Foodborne diseases – Waterborne diseases

ARTICLE SUMMARY

STRENGTHS AND LIMITATIONS OF THIS STUDY

- This prospective household study will provide detailed information on the incidence of, and risk factors for, secondary spread of cryptosporidiosis in the home
- This study will characterise *Cryptosporidium* isolates to ascertain likely mechanisms of spread by species
- This study will potentially identify the prevalence of asymptomatic infections with *Cryptosporidium*
- Common exposures across households may present problems with accurately identifying true secondary spread
- Biases may lead to a skewed sample of index cases because of under-ascertainment and bias in health-seeking behaviours

For peer review only - http://bmjopen.bmj.com/site/about/guidelines.xhtml

INTRODUCTION

Cryptosporidium is a protozoan parasite which can infect humans and other animals, and the most prevalent species identified in humans are *Cryptosporidium parvum* and *Cryptosporidium hominis* [1,2]. Cryptosporidiosis is the subsequent diarrhoeal disease following infection with *Cryptosporidium*. The disease affects all ages and although generally self-limiting, can be life threatening in some immune-compromised patients. Acute diarrhoea follows an incubation period of between 2-10 days (mean seven days) and symptoms can include non-bloody diarrhoea, abdominal cramps, vomiting and/or nausea, low grade fever, lethargy and general malaise.

Public Health England (PHE) receive laboratory reports of over 4,000 diagnosed cases per year (2000-2012 data) in England and Wales, however, research indicates that many infections may go undiagnosed, and the true incidence of disease may be much greater [3,4].

The parasite has a complex life cycle and characteristics which favour faecal-oral and environmental transmission routes, which may facilitate outbreaks via person-to-person (*C. hominis and C. parvum*) or animal-to-person (*C. parvum*) contact, as well as indirect transmission through ingestion of water and food contaminated with infectious oocysts [5].

Risk factors and associated exposures are often hypothesised/identified from outbreak investigations, however recognised outbreaks may only represent a small proportion of cases; estimates in the UK suggest, of all cases reported to national surveillance, <10% are likely to be linked to an identified outbreak [6] and contact with other people is highlighted as a factor in the transmission of *Cryptosporidium.* In a 1988 paper, onward transmission of *Cryptosporidium* was reported in households in the UK following a nursery outbreak, probably propagated by person-to-person spread in the home [7]. An analysis of outbreak reports from surveillance data in Ireland reported that ingestion of water and person-to-person spread were the most important mechanisms of transmission in outbreaks [8]. In the United States, in a case-control study evaluating sporadic cryptosporidiosis among immunocompetent persons, risk factors associated with increased odds of being a case

For peer review only - http://bmjopen.bmj.com/site/about/guidelines.xhtml

were international travel, contact with cattle, and contact with a child with diarrhoea [9]. In 2001-02, a case-control study conducted in the North West of England examined species-specific risk factors for sporadic cryptosporidiosis [10]. The authors compared risk factors for infection with genotypes 1 and 2 (currently recognised as *C. hominis and C. parvum* respectively) and found that contact with another person with diarrhoea was a risk factor for infection with *Cryptosporidium*, and that changing children's nappies was a specific risk factor for infection with *C. hominis* whether the child was symptomatic or not. Studies of *Giardia*, another gastrointestinal parasite, similar in terms of likely transmission routes, have recently been undertaken in the UK, and secondary spread and person-to-person transmission seems a likely and under-recognised route of transmission [11,12]. In a 1988 paper, onward transmission of *Cryptosporidium* was reported in households in the UK following a nursery outbreak, probably propagated by person-to-person spread in the home [9]. An analysis of outbreak reports from surveillance data in Ireland reported that ingestion of water and person-to-person spread were the most important mechanisms of transmission [10]. In the United States, a study evaluated sporadic cryptosporidiosis among immunocompetent persons using a case-control design. Risk factors associated with increased odds of being a case were international travel, contact with cattle, and contact with a child with diarrhoea. In 2001-02, a case-control study conducted in the North West of England examined species-specific risk factors for sporadic cryptosporidiosis. The authors compared risk factors for infection with genotypes 1 and 2 (currently recognised as *C. hominis and C. parvum* respectively) and found that contact with another person with diarrhoea was a risk factor for infection with *Cryptosporidium*, and that exposure through changing children's nappies was a specific risk factor for infection with *C. hominis* whether the child was symptomatic or not.

Asymptomatic spread

The burden of asymptomatic infection is less well documented in *Cryptosporidium* research than for other infections but may be an important factor in household spread. A study in the

For peer review only

UK reported a point prevalence of 1.3% among asymptomatic pre-school children attending day-care [13] suggesting that asymptomatic infection does occur. A Norwegian study looking at follow-on spread after two outbreaks found both asymptomatic and symptomatic infections in the households, which were likely to have been a result of secondary transmission [14]. Overall though, most of the work examining household spread has been undertaken in *Cryptosporidium*-endemic countries, where a high prevalence and repeated exposure to the organism might facilitate transmission, although immunity following repeat exposure is still poorly understood [15,16]. Newman *et al* undertook a prospective cohort study in Brazil to examine the transmission of *Cryptosporidium* infection in households where there was an identified case [17]. Secondary cases of infection occurred in 58% of households, and around a quarter of the identified secondary cases had diarrhoea, indicating the presence of asymptomatic infection in almost three-quarters of the participants. Similar results were reported from a longitudinal study in Bangladesh, where asymptomatic infection was more prevalent than diarrhoeal disease [18]. The same authors followed up with a case-control study in which the secondary attack rate was over 35%, and evidence of transmission in the home was further supported by genotyping results [19]. If person-to-person spread is driven by both cases and those with asymptomatic infections of *Cryptosporidium*, then sporadic cases may subsequently arise following exposure to either, and outbreaks in close settings such as the home or institutions may happen more frequently than is currently recognised. Few studies exist which refute or confirm this, especially in industrialised countries.

AIMS AND OBJECTIVES

The aim of this study is to estimate the amount of onward spread of *Cryptosporidium* that happens in the home, and to describe associated factors and case characteristics. (We use the term ‘secondary spread’ to mean any apparent onward transmission of disease originating from an index case, whilst recognising that this may be secondary or even tertiary levels of spread). This study will support our understanding of continued apparent sporadic cryptosporidiosis in England and Wales and has implications for appropriate public health

For peer review only

messages to help mitigate spread and infection. Further molecular characterisation of *Cryptosporidium* isolates may also help define the likelihood of secondary transmission by infecting species.

Objectives

- To estimate the number of secondary cases in households with an index case.
- To calculate the secondary transmission rate in households.
- To estimate the prevalence of asymptomatic carriage in households with an index case.
- To identify specific household-level and personal characteristics associated with secondary spread.
- To determine if factors and characteristics associated with secondary spread vary by species of *Cryptosporidium.*

METHODS

Study population

The study population will comprise residents of North West England and Wales. The North West of England has a population of over 7 million people and is the third-most populated region in the United Kingdom [20].. In 2016 over 600 laboratory-confirmed *Cryptosporidium* isolates were reported from the North West (8.4/100,000 population) [21].. Wales has a total population of over 3 million people [22]. In 2016, over 400 laboratory-confirmed *Cryptosporidium* isolates were reported from Wales, the highest rate of *Cryptosporidium spp* laboratory reports per 100,000 population in England and Wales (15/100,000) [21]..

Surveillance/sampling frame

The sampling frame will be taken from the two relevant surveillance systems which capture laboratory confirmed reports of *Cryptosporidium:* The Second-Generation Surveillance System (SGSS) in Public Heath England (PHE), and Tarian in Public Health Wales (PHW).

For peer review only

Systematic national surveillance of laboratory confirmed *Cryptosporidium* in England and Wales has been established for many years [23]. In the UK, *Cryptosporidium* is a notifiable causative agent, meaning laboratories have a statutory duty to notify the relevant public health authority of its identification in any human samples [24,25]. Cryptosporidiosis may present similarly to other causes of gastroenteritis, and laboratory confirmation of infection with *Cryptosporidium* is necessary for a diagnosis. Clinical practice may differ and clinicians would likely submit a sample to a primary diagnostic microbiology for a diagnosis of gastroenteritis. Local diagnostic laboratories across the UK use different methods to test for *Cryptosporidium*, and various criteria to decide whether to test for this parasite, including stool consistency, history or clinical details, duration of hospitalisation or clinician requests [26] Positive samples identified in the diagnostic laboratories are routinely forwarded to the national Cryptosporidium Reference Unit (CRU) which provides expert management, prevention and control advice as well as *Cryptosporidium* typing and confirmation services for speciation and surveillance [27].

All cases of laboratory confirmed *Cryptosporidium sp*. reported from primary diagnostic microbiology laboratories in North West England and Wales, in the study year, will initially be eligible.

Study type

The identification of cases, and their subsequent recruitment, is cross-sectional, although the study also involves retrospective data collection and some prospective sampling (**Error! Reference source not found.**).

Index cases of cryptosporidiosis will be identified via the relevant surveillance system(s). Once participants are recruited, they will complete a questionnaire (one per household), collecting clinical (onset date, symptoms of any household member, other illnesses of index case), demographic (age, sex, relationship to the index case), and household composition (type, number of bathrooms and bedrooms, animals) information. In addition, consenting household members (excluding the index case) will be asked to supply a stool sample.

For peer review only

Study period

The study period will be 12 months, to account for seasonal variation and allow maximum enrolment, up to 400 households. The study is expected to begin with a pilot phase of 1-2 months in autumn 2018.

Sample size

Given that the North West & Wales report around 1,000 cases per year (PHE data, 2015) and assuming a participation rate of 40-60%, and some exclusions (based on similar studies/approaches [11,28]) we anticipate a sample size of 400 households. Using 2011 Census indications of 2.4 persons on average per household [20], we can expect to recruit around 960 -1,000 participants in total. Assuming that the rate of household transmission, defined as the proportion of households with more than one case, is between 0% and 20% [11,14,17,29–31], a range of sample sizes was estimated (118-402). Recruitment of 400 households is feasible, and is sufficient to allow us to demonstrate a statistically significant minimum odds/risk ratio of 2.0, with type 1 error 0.05 and type 2 error at 0.20.

Case definition(s)

Tables 1 and 2 outline case and household definitions used to categorise household members and define secondary transmission.

For peer review only

For peer review only

Case definitions

Index case

The first case from a household identified in the surveillance system (person reported to a PHE/PHW surveillance system(s) following detection of *Cryptosporidium sp.* in a faecal sample, with a specimen date in the study year)

Secondary case(s)[1]

<u>Probable secondary case</u>

A person in a household of an index case, with symptoms:
of diarrhoea and/or vomiting
AND
that started after another case's onset date in the household

<u>Confirmed secondary case</u>

A person in a household of an index case, with symptoms:
of diarrhoea and/or vomiting
AND
That started after another case's onset date in the household;
AND
a *Cryptosporidium* positive stool sample

Asymptomatic

A person in a household of an index case with:
no reports of gastrointestinal illness;
AND
a *Cryptosporidium* positive stool sample.

[1] We use the term 'secondary spread' to mean any apparent onward transmission of disease originating from an index case, whilst recognising that this may be secondary or even tertiary levels of spread

For peer review only

277 *Table 1: Case definitions*

Household definitions
**Household**
Two or more people (not necessarily related) living at the same address in North West England or Wales who share cooking facilities and share a living room or sitting room or dining area [18].
**Household member**
A person who normally resides in the household and regularly shares food or toilet facilities [32]
**Household contact**
A household member where an index case has been identified
**Household with transmission**
A household that has more than one case
**Household without transmission**
A household that has one case (the index case)

278 *Table 2: Household definition*

For peer review only

Recruitment approach

Overview and rationale

Cases of cryptosporidiosis are identified from routine surveillance (from diagnostic laboratories) and are contacted via post, by the relevant public health organisation, in the first instance. Following this, if they do not opt-out, they are contacted via telephone by an NHS research nurse at the local Clinical Research Network (CRN) to chat about the study and determine if they would like to take part.

Our approach to the recruitment process was driven by necessity and feasibility and we explored several options at the protocol drafting stage of the project, balancing data needs with patient choice. As our capture of cases in the surveillance systems is retrospective and diagnosis of *Cryptosporidium* in the stool sample is undertaken by laboratory staff, there is no opportunity to consent individuals at the time of diagnosis and the recruitment process could not be achieved without access to patient information. In our model, participants are given opt-out options at each contact and it is emphasised that they can withdraw at any time. Previous research supports the acceptability and understanding of this method, recognising that an approach of 'consent for each use' is burdensome for both researcher and participant [33] [34], as does patient response and engagement with similar studies.[2]

Public and Patient Involvement

Patients and public were not involved in the overall design of the study, but we did elicit some public opinion when finalising our approach to recruitment. Following valuable comments from the ethical review board we undertook a short survey among the public and specific Patient and Public Involvement (PPI) groups to gauge general attitudes toward accessing data prior to consent, to support recruitment to research. We drafted a survey which outlined the approach to recruitment and the framework of the study. We accessed a lay PPI group from the Infection and Global Heath panel at the University of Liverpool, and

---

[2] Studies recruiting based on disease surveillance are common for GI infections, and many projects have taken this approach – the methodology for the epiCrypt Study has been influenced by design aspects of large-scale studies such as Enigma, IID2, and Integrate.

For peer review only

one from Health and Care Research Wales. Participants were asked to think generally about the method of recruitment and how they felt about this approach. In general, the feeling was that it is acceptable to access data for recruitment, especially to support much needed research. However, considerations and worries included the person accessing data, with NHS/public health staff generally viewed as more favourable that non-NHS (e.g. University) researchers.

Identification and first contact with the index case

Laboratory diagnosed reports of *Cryptosporidium*, and the corresponding patient contact details, will be extracted from the relevant surveillance system by health protection staff and saved in a line list (the Master copy of a confidential, separate table (MS Excel spreadsheet) holding patient details of all downloaded cases).

All potentially eligible participants will be issued a unique sequential study ID by PHE/PHW staff. This will be on all relevant study documentation and stool pots and follow each person and household through the study journey. This will allow data to be linked pseudonymously and helps with data management.

Staff from either PHE or PHW (depending on case location) will send an invite letter through the post to these potentially eligible index cases. The invite letter outlines the study, describes why the case has been contacted, and explains that a research nurse may be in touch over the coming weeks to discuss the study. The letter allows the case to choose several ways of opting-out of this contact (e-mail, freepost, telephone) and provides a named, clinical study lead for each public health organisation should they wish to discuss any aspect of this.

Approaching to recruit

If a contacted case does not opt-out within two weeks, their details will be shared securely (using internally agreed practices) with the NHS research nurses at the Clinical Research Network North West Coast (CRN). The research nurses will attempt to contact the index case (or parent/guardian of) via telephone (using internally agreed practices) to inform them about the study and offer them the opportunity to participate, if eligible. A maximum of three

For peer review only

332 attempts will be made, and nurses will not leave voicemails. If a case is unable to be contacted, or does not wish to participate at this stage, their details will be deleted from the line list. If the approached index case is successfully contacted via telephone and interested in participating, or would like more information, the research nurses will prepare and post a study pack. Index cases may be excluded at this stage where discussions with the case reveal that any of the following exclusion criteria apply:

- Index case is in a single-person household;
- Index case is a visitor to a household in the study area, but is registered with a GP outside the study area;
- Household is outside the study area;
- The index case is resident in an institution: retirement home, nursing home, prison, barracks, boarding school, or college/university halls of residence.

Study packs

The study packs contain:

- A study information pamphlet;
- A questionnaire booklet for the index case or a suitable representative (e.g. parent, head of household) to complete, with a freepost envelope;
- A consent form for each participating household member to read, initial, and sign (forms part of the questionnaire));
- A stool sampling pack (Fe-Col®) for each participating household member, with the required return postal envelope;
- An information leaflet on cryptosporidiosis and the relevant health advice;
- An information sheet on General Data Protection Regulation (GDPR) for health and care research.

Consent

For peer review only

The index case and any household members who wish to take part will sign and return the consent form at the front of the questionnaire. The return of study materials such as a completed questionnaire and/or stool samples will be considered implied consent.

Disenrollment

If study materials are not received within 14 days of posting the pack, a reminder letter will be dispatched by the research nurses at the CRN. If study documentation is not returned within 14 days of posting the reminder letter, no further attempt at contact will be made and the index case will be removed from the study line list.

Participation

If the household wants to take part, all interested members will sign and return the consent form. At least one household member, as well as the index case, must consent.

Consent forms and questionnaires are returned to the University of Liverpool in a stamped addressed envelope provided in the pack. The unique study IDs of those consenting will be shared weekly with the research nurses at the CRN to cross-match those contacted index cases that have been recruited and enrolled. Each consenting household member will also be asked to provide a stool sample for testing, using the provided Fe-Col® kits, which include a pre-addressed and secure postal bag (compliant with UN3373 regulations for mailing Cat B biological samples [35]). Instructions are provided, and samples will be returned directly to the *Cryptosporidium* Reference Unit.

Data management and oversight

Documentation

Questionnaire data will be inputted from the paper format to a corresponding MS Access database and held securely on a University of Liverpool drive in accordance with their security protocols. Figure 2 shows the data flow expected. Double data entry will be undertaken on a sample of questionnaires and discrepancies resolved using internal validation checks. When data entry is complete the data will be exported to the final study database [MS Access] where the data are pseudonymised for analyses: Name, date of birth,

For peer review only

and full postcode will be removed and replaced with unique study ID, age, and Lower Super Output Area (LSOAs are a type of geographic area in England and Wales, comprised of >1,000 residents [36]).

All data storage, cleaning and analyses will be undertaken by the study team at the University of Liverpool. Data will be stored on institutional network drives with appropriate security measures in place. Hard copy records will be stored in a locked cabinet in a secure location and access to records and data are limited to study personnel. Paper documents will be stored separately from the corresponding electronic data. The sponsor and data controller for this study is the Clinical Governance Team at the University of Liverpool.

Data security protocol available on request

Stool sample management

For purposes of data confidentiality and governance, stool samples returned to the *Cryptosporidium* Reference Unit are pseudonymised with the unique study ID, and participants will be asked to write their age, sex, and date of sample on their sample pot before they collect the stool. Stool sample results will be added to a study-specific database held at the CRU. The stool sample results will be added to the study database at the end of data collection using unique study ID in a secure file transfer.

Identifying the index case samples

Original diagnostic laboratory numbers will be retained with the index case information in the original line lists at PHE/PHW so that the sample can be identified later at the CRU and grouped with the relevant household samples. This sample, when located at the CRU, will be processed in the same way as other study samples.

Full laboratory protocol available on request

Outcomes and measurements

Questionnaire data

The questionnaire is divided into sections and is mostly composed of dichotomous and multiple-choice questions. Section A asks questions to determine the composition of the

For peer review only

household, the clinical details of the index case, and captures any other symptomatic household members. A table is used to collect information on any other symptomatic diarrhoeal illness in the house and will attempt to capture relationships to the index case (Table 3:).

Age	*Sex*	*Relationship to first case*	*Been ill with diarrhoea and/or vomiting* <br>*[Yes/No/Don't know]*	*When they became ill* <br>*[Date if known, otherwise before/after the first case]*	*How many days were they ill with these symptoms?*	*Did they see a doctor about this illness?*
39	*F*	*Mother*	*Yes*	*18/12/2017*	*10*	*No*
42	*M*	*Father*	*Yes*	*Before*	*About 3 days*	*No*
6 months	*M*	*Brother*	*No*	-		
24	*F*	*Lodger/House mate*	*Don't know*	-		

Table 3: An example table showing information collected in the questionnaire about other illness in the house

Section B records activities of the index case, and others in the home, in the two weeks prior to the index case's onset, based on known exposures for *Cryptosporidium*. Information on outdoor and leisure activities may help determine possible co-primary infections and distinguish them from those that are secondary. (Table 4:)

Sections C and D collect household variables, including the number of bedrooms and bathrooms, and capturing those who share beds/baths, and asking about outside space and animals. We also ask about nappy changing and toilet training in the home, and about general hand-washing behaviour.

Full questionnaire available on request.

For peer review only

Activity	FIRST CASE	ANYONE ELSE IN THE HOME
Travel outside the UK		
Swimming – outdoors in a lake, river, stream etc. (wild swimming)		
Swimming – in a treated swimming pool, either indoors or outdoors (such as a pool at a leisure centre or a lido)		
Other water activities/sport (such as surfing, rowing, water-skiing etc.)		
Other outdoor activities (such as camping, climbing, hiking, cycling etc.)		
Gardening (at home or elsewhere, such as an allotment)		
Contact with pets (at home or with pets at another house)		
Visiting or working on a farm or had contact with farm animals		
Visiting or working at a zoo or had contact with zoo or wild animals		

Table 4: An example table showing information collected in the questionnaire about activities

Stool collection and genotyping

All consenting household members of the index case (but not the index case) will be asked to provide a stool sample using the Fe-Col® kit (Figure 3) provided in the study pack, and post to the Cryptosporidium Reference Unit.

The stool pots will be labelled with the unique household ID which identifies them as part of the study but allows the samples to remain anonymous to the reference laboratory team. Samples will be scored against the Bristol stool scale and tested and quantified, only for *Cryptosporidium*, using immunofluorescence (IF) microscopy and real-time PCR. Positive samples will be speciated using validated PCR techniques which are part of normal practice [37]. *Cryptosporidium* DNA will be retained for subtyping and possibly whole genome sequencing (WGS) at a later date.

For peer review only

Full laboratory protocol available on request

ANALYSES

The primary objective of this study is to determine the amount of spread that happens in the home where there is a case of *Cryptosporidium*. This will be established by testing stool samples of household members of a case for *Cryptosporidium*, and reporting the numbers of secondary cases (according to our pre-determined definitions). A household with more than one case will be a household with secondary transmission.

We will calculate the following:

- The secondary transmission rate/prevalence within households (number of cases in the home/numbers at risk in the home, number of households with secondary spread /number of households));
- The amount of asymptomatic carriage among those exposed to symptomatic cases (number of asymptomatic cases/number at risk));
- Odds/Risk (OR/RR) of secondary illness according to activities and case/household characteristics;
- Odds/Risk (OR/RR) of secondary illness according to organism species.

Confounding (e.g. host factors such as age, co-morbidity) will be considered, where known, using multivariable techniques. Also, we will, where possible, examine environmental level exposures using stratification, e.g. those households/cases which are exposed to other known sources or risk factors, such as those living on farms.

Data will be analysed using Stata v12.

LIMITATIONS AND BIASES

Some elements of the study design are retrospective in nature, as the index case must have already been ill and been tested in order to be selected. As a result, some ascertainment bias may lead to a skewed sample from which to choose the index cases. We do not expect to capture the full profile of cases and households in the population that might have

For peer review only

472 *Cryptosporidium* due to differences in risk or vulnerability, severity, or health-seeking
473 behaviour[38]. We may get an over-representation of severe disease as these cases are
474 more likely to seek health care and be tested, and perhaps more likely to test positive.

475 We are only collecting one sample from each household member, and not re-sampling the
476 index case, for time and resource reasons. This may well lead to missing intermittent
477 shedding of oocysts, tertiary household infections and/or misclassifying recurring illness.

478 As *Cryptosporidium* is common in younger age groups, we expect a large proportion of the
479 participants to represent families with young children which may lead to over-representation
480 of these households. In addition, we might expect that having young children who were ill, or
481 being severely ill themselves, may incentivise cases to participate in the study, more than
482 adult, less severe cases.

483 Any likely over- or under-representation in the data collected will be considered when
484 assessing and describing results. Further unidentifiable limitations may include recall biases
485 around dates of onset or activities, and classification biases as we are asking about self-
486 reported illness and information may be inaccurate.

487 There is a possibility that we could see ongoing outbreaks in the study year. If this happens,
488 we might try to identify these where possible and may consider excluding households from
489 this study where all or most members have been exposed, including a definition of a co-
490 primary case.

491

492 **End of study**

493 The study will be declared as ended when the database is closed to recruitment – after one
494 year or when the maximum number of households has been enrolled.

495 **Pilot arrangements**

For peer review only

A pilot phase of one month is anticipated before data collection begins to assess and evaluate processes. Pilot data will be included as study data if no major methodological changes are proposed.

Ethics and dissemination

The study has been approved by the North West – Liverpool East NHS Research Ethics Committee (REC Reference: 18/NW/0300) and the Confidentiality and Advisory Group (Reference 18/CAG/0084). The project is registered on the National Institute for Health Research (NIHR) portfolio (CPMS ID – 39458).

Outputs will include scientific conferences and peer-reviewed publications. In addition, a short, lay report of findings will be produced for participants, who can opt to receive this when they take part.

Figure legend/caption

Fig 1: Outline of study

Fig 2: Study flow diagram

Fig 2: Fe-Col kit

References

1 Chalmers RM, Smith R, Elwin K, *et al.* Epidemiology of anthroponotic and zoonotic human cryptosporidiosis in England and Wales, 2004 to 2006. *Epidemiol Infect* 2011;**139**:700–12.http://www.jstor.org/stable/27975646

2 Cacciò SM, Putignani L. Epidemiology of Human Cryptosporidiosis. In: *Cryptosporidium: parasite and disease*. Vienna: : Springer Vienna 2014. 43–79. doi:10.1007/978-3-7091-1562-6_2

3 Adak GK, Long SM, O'Brien SJ. Trends in indigenous foodborne disease and deaths, England and Wales: 1992 to 2000. *Gut* 2002;**51**:832–41.http://www.ncbi.nlm.nih.gov/pubmed/12427786 (accessed 12 Jun 2017).

4 Food Standards Agency. A report of infectious intestinal disease in England. London: 2000.

For peer review only

5 Cacciò SM, Thompson RCA, McLauchlin J, *et al.* Unravelling Cryptosporidium and Giardia epidemiology. *Trends Parasitol* 2005;**21**:430–7. doi:10.1016/j.pt.2005.06.013

6 Chalmers RM, Giles M. Zoonotic cryptosporidiosis in the UK – challenges for control. *J Appl Microbiol* 2010;**109**:1487–97. doi:10.1111/j.1365-2672.2010.04764.x

7 Hannah J, Riordan T. Case to case spread of cryptosporidiosis; evidence from a day nursery outbreak. *Public Health* 1988;**102**:539–44.https://www.ncbi.nlm.nih.gov/pubmed/3231695

8 Garvey P, McKeown P. EPIDEMIOLOGY OF HUMAN CRYPTOSPORIDIOSIS IN IRELAND, 2004-2006: ANALYSIS OF NATIONAL NOTIFICATION DATA. *EUROSURVEILLANCE* 2009;**14**:16–20.

9 Abubakar I, Aliyu S, Arumugam C, *et al.* Risk factors for sporadic cryptosporidiosis among immunocompetent persons in the United States from 1999 to 2001. *J Clin Microbiol* 2004;**37**:2944–51. doi:10.3201/eid0905.020678

10 Hunter PR, Hughes S, Woodhouse S, *et al.* Sporadic cryptosporidiosis case-control study with genotyping. *Emerg Infect Dis* 2004;**10**:1241–9. doi:10.3201/eid1007.030582

11 Waldram A, Vivancos R, Hartley C, *et al.* Prevalence of Giardia infection in households of Giardia cases and risk factors for household transmission. *BMC Infect Dis* 2017;**17**:486. doi:10.1186/s12879-017-2586-3

12 Minetti C, Lamden K, Durband C, *et al.* Case-Control Study of Risk Factors for Sporadic Giardiasis and Parasite Assemblages in North West England. *J Clin Microbiol* 2015;**53**:3133–40. doi:10.1128/JCM.00715-15

13 Davies A, Campbell B, Evans MR, *et al.* Asymptomatic carriage of protozoan parasites in children in day care centers in the United Kingdom. *Pediatr Infect Dis J* 2009;**28**:838–40.https://insights.ovid.com/pubmed?pmid=19684527

14 Johansen OH, Hanevik K, Thrana F, *et al.* Symptomatic and asymptomatic secondary transmission of Cryptosporidium parvum following two related outbreaks in schoolchildren. *Epidemiol Infect* 2014;**143**:1–8. doi:10.1017/S095026881400243X

For peer review only

15 PR. H, RC. T. The zoonotic transmission of Giardia and Cryptosporidium. *Int J Parasitol* 2005;**35**:1181–90. doi:10.1016/j.ijpara.2005.07.009

16 Frost FJ, Roberts M, Kunde TR, *et al.* How Clean Must Our Drinking Water Be: The Importance of Protective Immunity. *J Infect Dis* 2005;**191**:809–14. doi:10.1086/427561

17 Newman RD, Zu SX, Wuhib T, *et al.* Household epidemiology of Cryptosporidium parvum infection in an urban community in Northeast Brazil. *Ann Intern Med* 1994;**120**:500–5.http://www.ncbi.nlm.nih.gov/pubmed/8311373 (accessed 27 Apr 2017).

18 Korpe PS, Haque R, Gilchrist C, *et al.* Natural History of Cryptosporidiosis in a Longitudinal Study of Slum-Dwelling Bangladeshi Children: Association with Severe Malnutrition. *PLoS Negl Trop Dis* 2016;**10**:e0004564. doi:10.1371/journal.pntd.0004564

19 Korpe PS, Gilchrist C, Burkey C, *et al.* Case-Control Study of Cryptosporidium Transmission in Bangladeshi Households. *Clin Infect Dis* Published Online First: 6 September 2018. doi:10.1093/cid/ciy593

20 ONS. 2011 Census - Office for National Statistics. https://www.ons.gov.uk/peoplepopulationandcommunity/populationandmigration/populationestimates/bulletins/populationandhouseholdestimatesfortheunitedkingdom/2011-03-21#number-of-people-in-households (accessed 11 Jun 2018).

21 PHE. Cryptosporidium: national laboratory data - GOV.UK. https://www.gov.uk/government/publications/cryptosporidium-national-laboratory-data (accessed 11 Jun 2018).

22 Welsh Government | Census of population. Stat. Res. 2015.https://gov.wales/statistics-and-research/census-population/?lang=en (accessed 10 Aug 2018).

23 Wall PG, de Louvois J, Gilbert RJ, *et al.* Food poisoning: notifications, laboratory reports, and outbreaks--where do the statistics come from and what do they mean?

For peer review only

Commun Dis Rep CDR Rev 1996;**6**:R93-100.http://www.ncbi.nlm.nih.gov/pubmed/8680502 (accessed 10 Aug 2018).

24 Public Health (Control of Disease) Act 1984. 1984.https://www.legislation.gov.uk/ukpga/1984/22 (accessed 10 Aug 2018).

25 The Health Protection (Notification) Regulations 2010. 2010.http://www.legislation.gov.uk/uksi/2010/659/regulation/4/made (accessed 10 Aug 2018).

26 Manuel R, Roche A, Young Y, *et al.* An audit of the laboratory diagnosis of cryptosporidiosis in England and Wales. *J Med Microbiol* 2015;**64**:688–93. doi:10.1099/jmm.0.000089

27 Public Health England. Cryptosporidium reference unit (CRU) - GOV.UK. https://www.gov.uk/guidance/cryptosporidium-reference-unit-cru (accessed 10 Aug 2018).

28 Tam CC, Rodrigues LC, Viviani L, *et al.* Longitudinal study of infectious intestinal disease in the UK (IID2 study): incidence in the community and presenting to general practice. *Gut* 2012;**61**:69–77. doi:10.1136/gut.2011.238386

29 Perry S, De La Luz Sanchez M, Hurst PK, *et al.* Household transmission of gastroenteritis. *Emerg Infect Dis* 2005;**11**:1093–6. doi:10.3201/eid1107.040889

30 Harrison SL, Nelder R, Hayek L, *et al.* Managing a large outbreak of cryptosporidiosis: how to investigate and when to decide to lift a 'boil water' notice. *Commun Dis public Heal* 2002;**5**:230–9.

31 MacKenzie WR, Schell WL, Blair KA, *et al.* Massive outbreak of waterborne cryptosporidium infection in Milwaukee, Wisconsin: recurrence of illness and risk of secondary transmission. *Clin Infect Dis* 1995;**21**:57–62.http://www.ncbi.nlm.nih.gov/pubmed/7578760 (accessed 27 Apr 2017).

32 Public health control and management of hepatitis A 2017 Guidelines. Published Online First: 2017.https://www.gov.uk/government/uploads/system/uploads/attachment_data/file/6

For peer review only

23036/Public_health_control_and_management_of_hepatitis_A_2017.pdf (accessed 30 Oct 2017).

33 Willison DJ, Swinton M, Schwartz L, *et al.* Alternatives to project-specific consent for access to personal information for health research: insights from a public dialogue. *BMC Med Ethics* 2008;**9**:18. doi:10.1186/1472-6939-9-18

34 Taylor MJ, Taylor N. Health research access to personal confidential data in England and Wales: assessing any gap in public attitude between preferable and acceptable models of consent. *Life Sci Soc policy* 2014;**10**:15. doi:10.1186/s40504-014-0015-6

35 Category B - Medical Packaging, medical pouches and biological substances on un3373.com. http://www.un3373.com/category-biological-substances/category-b/ (accessed 10 Aug 2018).

36 Office for National Statistics. Overview of the Various Geographies Used in the Production of Statistics Collected via the UK Census.

37 Robinson G EK and CR. Methods and Protocols in Cryptosporidium Research. Cryptosporidium diagnostic assays: molecular detection. In: Ed. Mead J, ed. *Methods in Molecular Biology*. Springer Nature

38 Adams NL, Rose TC, Hawker J, *et al.* Socioeconomic status and infectious intestinal disease in the community: a longitudinal study (IID2 study). *Eur J Public Health* 2018;**28**:134–8. doi:10.1093/eurpub/ckx091

For peer review only

Authors' contributions

CMCK, RCha, RV, RChr, SOB conceived the initial idea for the study and the methodology. JM contributed to methods for participant recruitment and ethics submissions. CMCK wrote the protocol. RChr, RCha, RV, SOB reviewed and revised the protocol and the paper. All authors have read and approved the final manuscript.

Funding statement

This research is jointly funded by the National Institute for Health Research Health Protection Research Unit (NIHR HPRU) in Emerging and Zoonotic Infections (HPRU EZI)

and the National Institute for Health Research Health Protection Research Unit (NIHR HPRU) in Gastrointestinal Infections (HPRU GI) at the University of Liverpool in partnership with Public Health England (PHE), University of East Anglia, University of Oxford and the Institute of Food Research. The views expressed are those of the authors and not necessarily those of the NHS, the NIHR, the Department of Health, PHE or Public Health Wales.

Competing interests statement

The authors declare that they have no competing interests.

Acknowledgements

John Harris, Goutam Adak, Ken Lamden, Andrew Fox

The Clinical Research Network North West Coast, Liverpool

Laura Evans, Public Health Wales

Christopher Williams and the Health Protection Team, Public Health Wales

Jo Hardstaff, University of Liverpool

Richard Dunn and Elaine Mooney, Field Services, Public Health England

Infection and Global Heath panel (PPI group) at the University of Liverpool, Health and Care Research Wales, and all other public/lay contributors to the Public and Patient Involvement survey

For peer review only

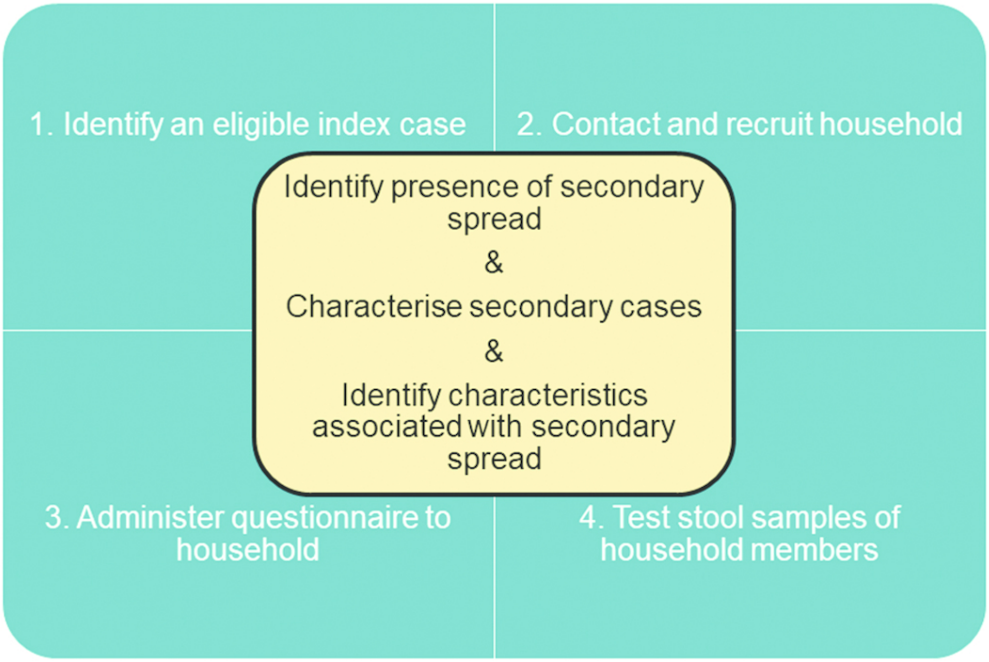

Outline of study

90x90mm (300 x 300 DPI)

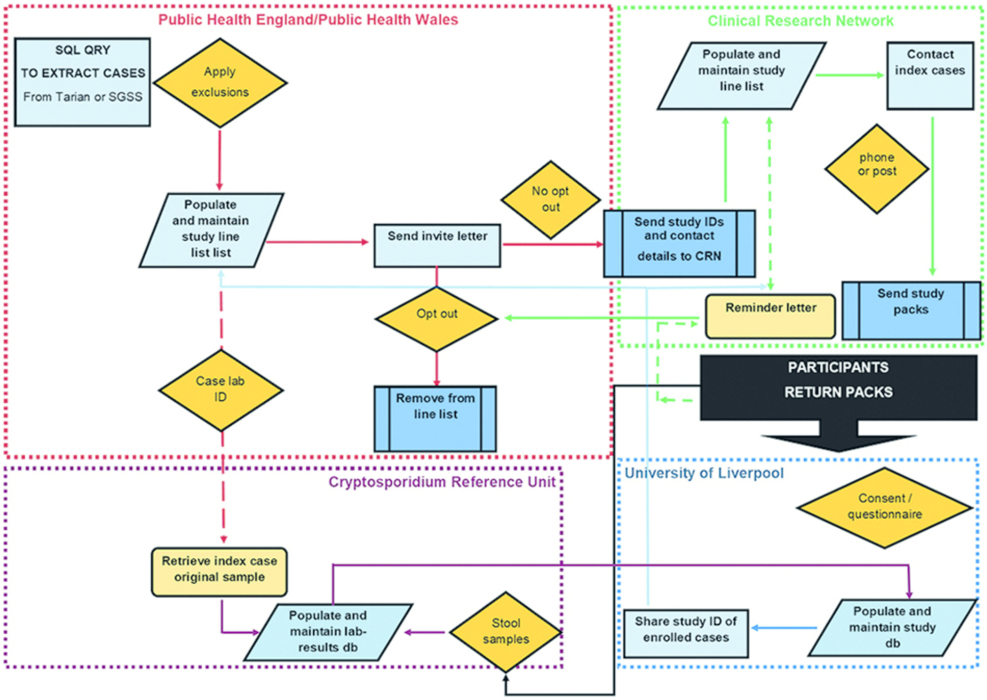

Study Flow Diagram

90x90mm (300 x 300 DPI)

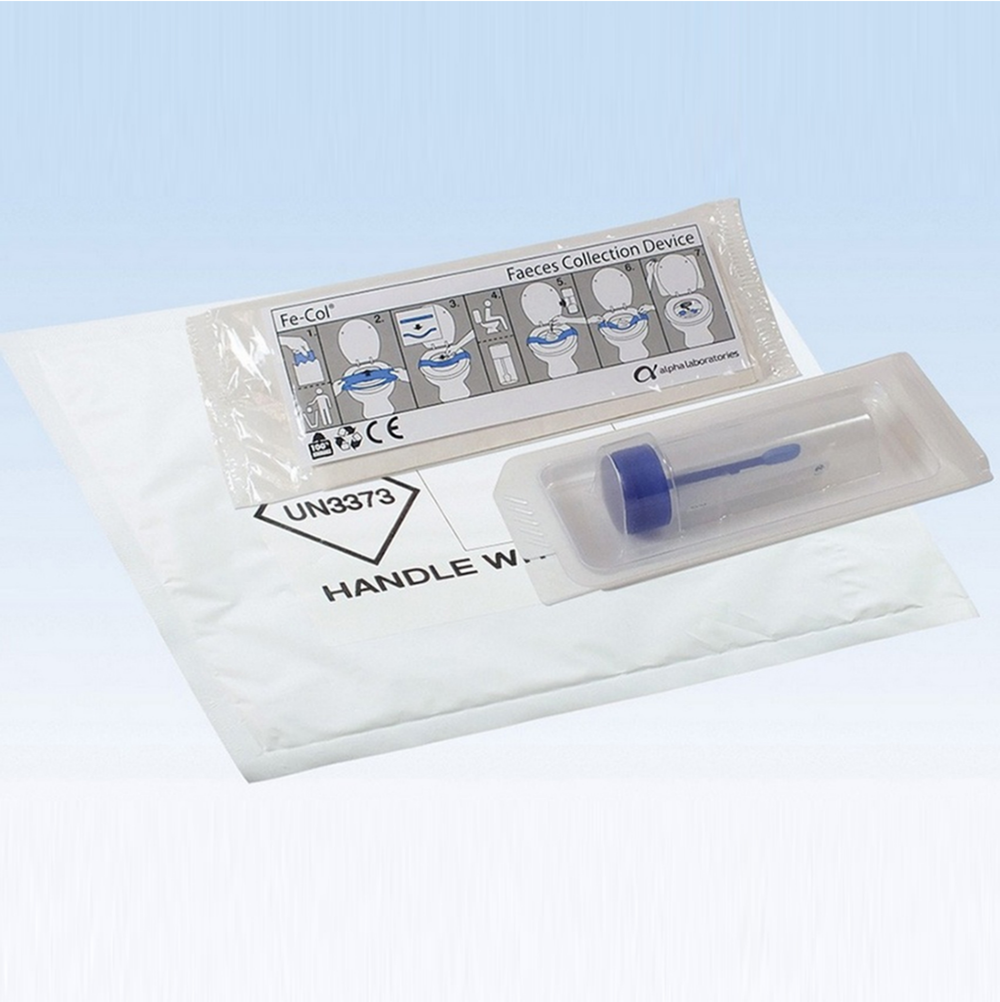

Fe-Col kit

90x90mm (300 x 300 DPI)

STROBE Statement—checklist of items that should be included in reports of observational studies

	Item No	Recommendation
**Title and abstract**	1	(*a*) Indicate the study's design with a commonly used term in the title or the abstract
		(*b*) Provide in the abstract an informative and balanced summary of what was done and what was found
**Introduction**		
Background/rationale	2	Explain the scientific background and rationale for the investigation being reported
Objectives	3	State specific objectives, including any prespecified hypotheses
**Methods**		
Study design	4	Present key elements of study design early in the paper
Setting	5	Describe the setting, locations, and relevant dates, including periods of recruitment, exposure, follow-up, and data collection
Participants	6	(*a*) *Cohort study*—Give the eligibility criteria, and the sources and methods of selection of participants. Describe methods of follow-up<br>*Case-control study*—Give the eligibility criteria, and the sources and methods of case ascertainment and control selection. Give the rationale for the choice of cases and controls<br>*Cross-sectional study*—Give the eligibility criteria, and the sources and methods of selection of participants
		(*b*) *Cohort study*—For matched studies, give matching criteria and number of exposed and unexposed<br>*Case-control study*—For matched studies, give matching criteria and the number of controls per case
Variables	7	Clearly define all outcomes, exposures, predictors, potential confounders, and effect modifiers. Give diagnostic criteria, if applicable
Data sources/ measurement	8*	For each variable of interest, give sources of data and details of methods of assessment (measurement). Describe comparability of assessment methods if there is more than one group
Bias	9	Describe any efforts to address potential sources of bias
Study size	10	Explain how the study size was arrived at
Quantitative variables	11	Explain how quantitative variables were handled in the analyses. If applicable, describe which groupings were chosen and why
Statistical methods	12	(*a*) Describe all statistical methods, including those used to control for confounding we are calculating ORs and percentages. Have outlined objectives.
		(*b*) Describe any methods used to examine subgroups and interactions
		(*c*) Explain how missing data were addressed
		(*d*) *Cohort study*—If applicable, explain how loss to follow-up was addressed<br>*Case-control study*—If applicable, explain how matching of cases and controls was addressed<br>*Cross-sectional study*—If applicable, describe analytical methods taking account of sampling strategy
		(*e*) Describe any sensitivity analyses

Comment [cmck1]: These may not be known until after the study data are analysed.
This is a protocol paper.
We do describe that we will use stratification for sub-groups if data are relevant.

Continued on next page

For peer review only

Comment [cmck2]: N/A Protocol

**Results n/a**		
Participants	13*	(a) Report numbers of individuals at each stage of study—eg numbers potentially eligible, examined for eligibility, confirmed eligible, included in the study, completing follow-up, and analysed
		(b) Give reasons for non-participation at each stage
		(c) Consider use of a flow diagram
Descriptive data	14*	(a) Give characteristics of study participants (eg demographic, clinical, social) and information on exposures and potential confounders
		(b) Indicate number of participants with missing data for each variable of interest
		(c) *Cohort study*—Summarise follow-up time (eg, average and total amount)
Outcome data	15*	*Cohort study*—Report numbers of outcome events or summary measures over time
		Case-control study—Report numbers in each exposure category, or summary measures of exposure
		Cross-sectional study—Report numbers of outcome events or summary measures
Main results	16	(*a*) Give unadjusted estimates and, if applicable, confounder-adjusted estimates and their precision (eg, 95% confidence interval). Make clear which confounders were adjusted for and why they were included
		(*b*) Report category boundaries when continuous variables were categorized
		(*c*) If relevant, consider translating estimates of relative risk into absolute risk for a meaningful time period
Other analyses	17	Report other analyses done—eg analyses of subgroups and interactions, and sensitivity analyses
**Discussion**		
Key results	18	Summarise key results with reference to study objectives
Limitations	19	Discuss limitations of the study, taking into account sources of potential bias or imprecision. Discuss both direction and magnitude of any potential bias
Interpretation	20	Give a cautious overall interpretation of results considering objectives, limitations, multiplicity of analyses, results from similar studies, and other relevant evidence
Generalisability	21	Discuss the generalisability (external validity) of the study results
**Other information**		
Funding	22	Give the source of funding and the role of the funders for the present study and, if applicable, for the original study on which the present article is based

*Give information separately for cases and controls in case-control studies and, if applicable, for exposed and unexposed groups in cohort and cross-sectional studies.

Note: An Explanation and Elaboration article discusses each checklist item and gives methodological background and published examples of transparent reporting. The STROBE checklist is best used in conjunction with this article (freely available on the Web sites of PLoS Medicine at http://www.plosmedicine.org/, Annals of Internal Medicine at http://www.annals.org/, and Epidemiology at http://www.epidem.com/). Information on the STROBE Initiative is available at www.strobe-statement.org.

For peer review only - http://bmjopen.bmj.com/site/about/guidelines.xhtml

BMJ Open

The epiCrypt study protocol: a cross-sectional investigation of household transmission of Cryptosporidium in England and Wales

Journal:	*BMJ Open*
Manuscript ID	bmjopen-2018-026116.R2
Article Type:	Protocol
Date Submitted by the Author:	08-Apr-2019

Complete List of Authors:	McKerr, Caoimhe; university of liverpool, NIHR Health Protection Research Unit in Gastrointestinal Infections; University of Liverpool, NIHR Health Protection Research Unit in Emerging and Zoonotic Infections<br>Chalmers, Rachel; Public Health Wales, Cryptosporidium Reference Unit; Swansea University Medical School<br>Vivancos, Roberto; Public Health England, Field Epidemiology Services, Health Protection; The University of Liverpool, NIHR Health Protection Research Unit in Emerging and Zoonotic Infections<br>O'Brien, Sarah; University of Liverpool, Institute of Psychology, Health and Society; University of Liverpool, NIHR Health Protection Research Unit in Gastrointestinal Infections<br>Mugarza, Julie; NIHR Clinical Research Network North West Coast<br>Christley, Robert; University of Liverpool, Department of Epidemiology and Population Health, Institute of Infection and Global Health; NIHR Health Protection Research Unit in Emerging and Zoonotic Infections

**Primary Subject Heading**:	Epidemiology
Secondary Subject Heading:	Epidemiology, Infectious diseases
Keywords:	Cryptosporidium, Household transmission, Secondary spread, Zoonoses, Gastrointestinal infection, Epidemiology < TROPICAL MEDICINE

SCHOLARONE™
Manuscripts

Short title

The epiCrypt Study - protocol

Word Count

≈4100 (excl. figs, tbls, refs)

Authors:

Caoimhe McKerr[1,2,6] / c.mckerr@liv.ac.uk

Rachel M Chalmers[3,7] / rachel.chalmers@wales.nhs.uk

Roberto Vivancos[1,2,4] / roberto.vivancos@phe.gov.uk

Sarah J O'Brien[1] / sjobrien@liv.ac.uk

Julie Mugarza[5] / julie.mugarza@nihr.ac.uk

Robert M Christley[2,6] / robc@liv.ac.uk

1 NIHR Health Protection Research Unit in Gastrointestinal Infections, The University of Liverpool

2 NIHR Health Protection Research Unit in Emerging and Zoonotic Infections, The University of Liverpool

3 *Cryptosporidium* Reference Unit, Public Health Wales, Swansea

4 Field Epidemiology Services, Public Health England, Liverpool

5 NIHR Clinical Research Network (CRN) North West Coast, Liverpool

6 Institute of Infection and Global Health, University of Liverpool

7 Swansea Medical School, Swansea University

For peer review only

Corresponding author:

Caoimhe McKerr

Current address:

The University of Liverpool

Institute of Infection and Global Health

Waterhouse Building (2nd Floor, Block F)

1-5 Brownlow Street

Liverpool L69 3GL

Email: c.mckerr@liverpool.ac.uk

For peer review only

TITLE

The epiCrypt study protocol: a cross-sectional investigation of household transmission of *Cryptosporidium* in England and Wales

AUTHORS

Caoimhe McKerr[1,2,6] / c.mckerr@liv.ac.uk

Rachel M Chalmers[3,7] / rachel.chalmers@wales.nhs.uk

Roberto Vivancos[1,2,4] / roberto.vivancos@phe.gov.uk

Sarah J O'Brien[1] / sjobrien@liv.ac.uk

Julie Mugarza[5] / julie.mugarza@nihr.ac.uk

Robert M Christley[2,6] / robc@liv.ac.uk

1 NIHR Health Protection Research Unit in Gastrointestinal Infections, The University of Liverpool

2 NIHR Health Protection Research Unit in Emerging and Zoonotic Infections, The University of Liverpool

3 *Cryptosporidium* Reference Unit, Public Health Wales, Swansea

4 Field Epidemiology Services, Public Health England, Liverpool

5 NIHR Clinical Research Network (CRN) North West Coast, Liverpool

6 Institute of Infection and Global Health, University of Liverpool

7 Swansea Medical School, Swansea University

Correspondence to: c.mckerr@liv.ac.uk

For peer review only

ABSTRACT

Introduction

Infection with the *Cryptosporidium* parasite causes over 4,000 cases of diagnosed illness (cryptosporidiosis) in England and Wales each year. Risk factors are often estimated from outbreak investigations, and in the UK include ingestion of contaminated water and food, farm/animal contact and person-to-person spread in institutions. However, reported outbreaks only represent about 10% of cases and the transmission routes for sporadic disease may not be the same. Contact with other people has been highlighted as a factor in the transmission of *Cryptosporidium*, but the incidence of sporadic disease has not been sufficiently established, and how frequently this arises from contact with other infected people is not well documented.

This project will estimate the amount of secondary spread that occurs in the home and potentially identify asymptomatic infections which might have a role in transmission. Risk factors and characteristics associated with secondary spread will be described including any differences in transmission between *Cryptosporidium* species.

Methods and analysis

The study will prospectively identify cryptosporidiosis cases from North West England and Wales over one year and invite them and their household to take part. Each household will complete a questionnaire and each household member will be asked to provide a stool sample. Clinical, demographic, and home variables will be described, and further analyses undertaken to investigate associations with secondary spread in the home.

Cryptosporidium-positive stool samples, identified by immunofluorescence microscopy, will be characterised using molecular methods to describe patterns of transmission. Data collection is expected to take one year, beginning in September 2018.

Ethics

The study has been approved by the North West – Liverpool East NHS Research Ethics Committee (Reference: 18/NW/0300) and the Confidentiality and Advisory Group (Reference

For peer review only

18/CAG/0084). The project is registered on the National Institute for Health Research (NIHR) portfolio (CPMS ID: 39458).

KEYWORDS

Cryptosporidium – Protozoa – Sporadic disease – Household transmission – Person-to-person – Secondary spread - Zoonoses – Gastrointestinal infection – Risk factors – Epidemiology - Parasite – Foodborne diseases – Waterborne diseases

ARTICLE SUMMARY

STRENGTHS AND LIMITATIONS OF THIS STUDY

- This prospective household study will provide detailed information on the incidence of, and risk factors for, secondary spread of cryptosporidiosis in the home
- This study will characterise *Cryptosporidium* isolates to ascertain likely mechanisms of spread by species
- This study will potentially identify the prevalence of asymptomatic infections with *Cryptosporidium*
- Common exposures across households may present problems with accurately identifying true secondary spread
- Biases may lead to a skewed sample of index cases because of under-ascertainment and bias in health-seeking behaviours

For peer review only - http://bmjopen.bmj.com/site/about/guidelines.xhtml

INTRODUCTION

Cryptosporidium is a protozoan parasite which can infect humans and other animals, and the most prevalent species identified in humans are *Cryptosporidium parvum* and *Cryptosporidium hominis* [1,2]. Cryptosporidiosis is the subsequent diarrhoeal disease following infection with *Cryptosporidium*. The disease affects all ages and although generally self-limiting, can be life threatening in some immune-compromised patients. Acute diarrhoea follows an incubation period of between 2-10 days (mean seven days) and symptoms can include non-bloody diarrhoea, abdominal cramps, vomiting and/or nausea, low grade fever, lethargy and general malaise.

Public Health England (PHE) receive laboratory reports of over 4,000 diagnosed cases per year (2000-2012 data) in England and Wales, however, research indicates that many infections may go undiagnosed, and the true incidence of disease may be much greater [3,4].

The parasite has a complex life cycle and characteristics which favour faecal-oral and environmental transmission routes, which may facilitate outbreaks via person-to-person (*C. hominis and C. parvum*) or animal-to-person (*C. parvum*) contact, as well as indirect transmission through ingestion of water and food contaminated with infectious oocysts [5].

Risk factors and associated exposures are often hypothesised/identified from outbreak investigations, however recognised outbreaks may only represent a small proportion of cases; estimates in the UK suggest, of all cases reported to national surveillance, <10% are likely to be linked to an identified outbreak [6] and contact with other people is highlighted as a factor in the transmission of *Cryptosporidium.* In a 1988 paper, onward transmission of *Cryptosporidium* was reported in households in the UK following a nursery outbreak, probably propagated by person-to-person spread in the home [7]. An analysis of outbreak reports from surveillance data in Ireland reported that ingestion of water and person-to-person spread were the most important mechanisms of transmission in outbreaks [8]. In the United States, in a case-control study evaluating sporadic cryptosporidiosis among immunocompetent persons, risk factors associated with increased odds of being a case

For peer review only

were international travel, contact with cattle, and contact with a child with diarrhoea [9]. In 2001-02, a case-control study conducted in the North West of England examined species-specific risk factors for sporadic cryptosporidiosis [10]. The authors compared risk factors for infection with genotypes 1 and 2 (currently recognised as *C. hominis and C. parvum* respectively) and found that contact with another person with diarrhoea was a risk factor for infection with *Cryptosporidium*, and that changing children's nappies was a specific risk factor for infection with *C. hominis* whether the child was symptomatic or not. Studies of *Giardia*, another gastrointestinal parasite, similar in terms of likely transmission routes, have recently been undertaken in the UK, and secondary spread and person-to-person transmission seems a likely and under-recognised route of transmission [11,12]. In a 1988 paper, onward transmission of *Cryptosporidium* was reported in households in the UK following a nursery outbreak, probably propagated by person-to-person spread in the home [9]. An analysis of outbreak reports from surveillance data in Ireland reported that ingestion of water and person-to-person spread were the most important mechanisms of transmission [10]. In the United States, a study evaluated sporadic cryptosporidiosis among immunocompetent persons using a case-control design. Risk factors associated with increased odds of being a case were international travel, contact with cattle, and contact with a child with diarrhoea. In 2001-02, a case-control study conducted in the North West of England examined species-specific risk factors for sporadic cryptosporidiosis. The authors compared risk factors for infection with genotypes 1 and 2 (currently recognised as *C. hominis and C. parvum* respectively) and found that contact with another person with diarrhoea was a risk factor for infection with *Cryptosporidium*, and that exposure through changing children's nappies was a specific risk factor for infection with *C. hominis* whether the child was symptomatic or not.

Asymptomatic spread

The burden of asymptomatic infection is less well documented in *Cryptosporidium* research than for other infections but may be an important factor in household spread. A study in the

For peer review only

UK reported a point prevalence of 1.3% among asymptomatic pre-school children attending day-care [13] suggesting that asymptomatic infection does occur. A Norwegian study looking at follow-on spread after two outbreaks found both asymptomatic and symptomatic infections in the households, which were likely to have been a result of secondary transmission [14]. Overall though, most of the work examining household spread has been undertaken in *Cryptosporidium*-endemic countries, where a high prevalence and repeated exposure to the organism might facilitate transmission, although immunity following repeat exposure is still poorly understood [15,16]. Newman *et al* undertook a prospective cohort study in Brazil to examine the transmission of *Cryptosporidium* infection in households where there was an identified case [17]. Secondary cases of infection occurred in 58% of households, and around a quarter of the identified secondary cases had diarrhoea, indicating the presence of asymptomatic infection in almost three-quarters of the participants. Similar results were reported from a longitudinal study in Bangladesh, where asymptomatic infection was more prevalent than diarrhoeal disease [18]. The same authors followed up with a case-control study in which the secondary attack rate was over 35%, and evidence of transmission in the home was further supported by genotyping results [19]. If person-to-person spread is driven by both cases and those with asymptomatic infections of *Cryptosporidium*, then sporadic cases may subsequently arise following exposure to either, and outbreaks in close settings such as the home or institutions may happen more frequently than is currently recognised. Few studies exist which refute or confirm this, especially in industrialised countries.

AIMS AND OBJECTIVES

The aim of this study is to estimate the amount of onward spread of *Cryptosporidium* that happens in the home, and to describe associated factors and case characteristics. (We use the term 'secondary spread' to mean any apparent onward transmission of disease originating from a case, whilst recognising that this may be secondary or even tertiary levels of spread). This study will support our understanding of continued apparent sporadic cryptosporidiosis in England and Wales and has implications for appropriate public health

For peer review only

messages to help mitigate spread and infection. Further molecular characterisation of *Cryptosporidium* isolates may also help define the likelihood of secondary transmission by infecting species.

Objectives

- To estimate the number of secondary cases in households with an index case.
- To calculate the secondary transmission rate in households.
- To estimate the prevalence of asymptomatic carriage in households with an index case.
- To identify specific household-level and personal characteristics associated with secondary spread.
- To determine if factors and characteristics associated with secondary spread vary by species of *Cryptosporidium.*

METHODS

Study population

The study population will comprise residents of North West England and Wales.
The North West of England has a population of over 7 million people and is the third-most populated region in the United Kingdom [20]. In 2016 over 600 laboratory-confirmed *Cryptosporidium* isolates were reported from the North West (8.4/100,000 population) [21]. Wales has a total population of over 3 million people [22]. In 2016, over 400 laboratory-confirmed *Cryptosporidium* isolates were reported from Wales, the highest rate of *Cryptosporidium spp* laboratory reports per 100,000 population in England and Wales (15/100,000) [21].

Surveillance/sampling frame

The sampling frame will be taken from the two relevant surveillance systems which capture laboratory confirmed reports of *Cryptosporidium:* The Second-Generation Surveillance System (SGSS) in Public Heath England (PHE), and Tarian in Public Health Wales (PHW).

For peer review only

Systematic national surveillance of laboratory confirmed *Cryptosporidium* in England and Wales has been established for many years [23]. In the UK, *Cryptosporidium* is a notifiable causative agent, meaning laboratories have a statutory duty to notify the relevant public health authority of its identification in any human samples [24,25]. Cryptosporidiosis may present similarly to other causes of gastroenteritis, and laboratory confirmation of infection with *Cryptosporidium* is necessary for a diagnosis. Clinical practice may differ, and clinicians would likely submit a sample to a primary diagnostic microbiology for a diagnosis of gastroenteritis. Local diagnostic laboratories across the UK use different methods to test for *Cryptosporidium*, and various criteria to decide whether to test for this parasite, including stool consistency, history or clinical details, duration of hospitalisation or clinician requests [26]. Positive samples identified in the diagnostic laboratories are routinely forwarded to the national Cryptosporidium Reference Unit (CRU) which provides expert management, prevention and control advice as well as *Cryptosporidium* typing and confirmation services for speciation and surveillance [27].

All cases of laboratory confirmed *Cryptosporidium sp*. reported from primary diagnostic microbiology laboratories in North West England and Wales, in the study year, will initially be eligible.

Study type

The identification of cases, and their subsequent recruitment, is cross-sectional, although the study also involves retrospective data collection and some prospective sampling (**Error! Reference source not found.**).

Cases of cryptosporidiosis will be identified via the relevant surveillance system(s). Once participants are recruited, they will complete a questionnaire (one per household), collecting clinical (onset date, symptoms of any household member, other illnesses of index case), demographic (age, sex, relationship to the index case), and household composition (type, number of bathrooms and bedrooms, animals) information. In addition, consenting household members (excluding the index case) will be asked to supply a stool sample.

For peer review only

Study period

The study period will be 12 months, to account for seasonal variation and allow maximum enrolment, up to 400 households. The study is expected to begin with a pilot phase of 1-2 months in autumn 2018.

Sample size

Given that the North West & Wales report around 1,000 cases per year (PHE data, 2015) and assuming a participation rate of 40-60%, and some exclusions (based on similar studies/approaches [11,28]) we anticipate a sample size of 400 households. Using 2011 Census indications of 2.4 persons on average per household [20], we can expect to recruit around 960 -1,000 participants in total. Assuming that the rate of household transmission, defined as the proportion of households with more than one case, is between 0% and 20% [11,14,17,29–31], a range of sample sizes was estimated (118-402). Recruitment of 400 households is feasible and is sufficient to allow us to demonstrate a statistically significant minimum odds/risk ratio of 2.0, with type 1 error 0.05 and type 2 error at 0.20.

Case definition(s)

Tables 1 and 2 outline case and household definitions used to categorise household members and define secondary transmission.

For peer review only

For peer review only

Case definitions

Index case
The first case from a household identified in the surveillance system (person reported to a PHE/PHW surveillance system(s) following detection of *Cryptosporidium sp.* in a faecal sample, with a specimen date in the study year)

Household case
Any household member of a index case who reports symptoms consistent with *Cryptosporidium* (diarrhoea and/or vomiting) and/or has a *Cryptosporidium* positive stool sample

Secondary case(s)[1]
<u>Probable secondary case</u>
A person in a household of an index case, with symptoms:
of diarrhoea and/or vomiting
AND
that started after another case's onset date in the household
<u>Confirmed secondary case</u>
A person in a household of an index case, with symptoms:
of diarrhoea and/or vomiting
AND
That started after another case's onset date in the household;
AND
a *Cryptosporidium* positive stool sample
Asymptomatic
A person in a household of an index case with:

[1] We use the term 'secondary spread' to mean any apparent onward transmission of disease originating from an index case, whilst recognising that this may be secondary or even tertiary levels of spread

For peer review only

no reports of gastrointestinal illness;<br>AND<br>a *Cryptosporidium* positive stool sample.

Table 1: Case definitions

Household definitions
**Household**<br>Two or more people (not necessarily related) living at the same address in North West England or Wales who share cooking facilities and share a living room or sitting room or dining area [18].
**Household member**<br>A person who normally resides in the household and regularly shares food or toilet facilities [32]
**Household contact**<br>A household member where an index case has been identified
**Household with transmission**<br>A household that has more than one case
**Household without transmission**<br>A household that has one case (the index case)

Table 2: Household definition

For peer review only

Recruitment approach

Overview and rationale

Cases of cryptosporidiosis are identified from routine surveillance (from diagnostic laboratories) and are contacted via post, by the relevant public health organisation, in the first instance. Following this, if they do not opt-out, they are contacted via telephone by an NHS research nurse at the local Clinical Research Network (CRN) to chat about the study and determine if they would like to take part.

Our approach to the recruitment process was driven by necessity and feasibility and we explored several options at the protocol drafting stage of the project, balancing data needs with patient choice. As our capture of cases in the surveillance systems is retrospective and diagnosis of *Cryptosporidium* in the stool sample is undertaken by laboratory staff, there is no opportunity to consent individuals at the time of diagnosis and the recruitment process could not be achieved without access to patient information. In our model, participants are given opt-out options at each contact and it is emphasised that they can withdraw at any time. Previous research supports the acceptability and understanding of this method, recognising that an approach of 'consent for each use' is burdensome for both researcher and participant [33] [34], as does patient response and engagement with similar studies.[2]

Public and Patient Involvement

Patients and public were not involved in the overall design of the study, but we did elicit some public opinion when finalising our approach to recruitment. Following valuable comments from the ethical review board we undertook a short survey among the public and specific Patient and Public Involvement (PPI) groups to gauge general attitudes toward accessing data prior to consent, to support recruitment to research. We drafted a survey which outlined the approach to recruitment and the framework of the study. We accessed a lay PPI group from the Infection and Global Heath panel at the University of Liverpool, and

[2] Studies recruiting based on disease surveillance are common for GI infections, and many projects have taken this approach – the methodology for the epiCrypt Study has been influenced by design aspects of large-scale studies such as Enigma, IID2, and Integrate.

For peer review only - http://bmjopen.bmj.com/site/about/guidelines.xhtml

one from Health and Care Research Wales. Participants were asked to think generally about the method of recruitment and how they felt about this approach. In general, the feeling was that it is acceptable to access data for recruitment, especially to support much needed research. However, considerations and worries included the person accessing data, with NHS/public health staff generally viewed as more favourable that non-NHS (e.g. University) researchers.

Identification and first contact with the index case

Laboratory diagnosed reports of *Cryptosporidium*, and the corresponding patient contact details, will be extracted from the relevant surveillance system by health protection staff and saved in a line list (the Master copy of a confidential, separate table (MS Excel spreadsheet) holding patient details of all downloaded cases).

All potentially eligible participants will be issued a unique sequential study ID by PHE/PHW staff. This will be on all relevant study documentation and stool pots and follow each person and household through the study journey. This will allow data to be linked pseudonymously and helps with data management.

Staff from either PHE or PHW (depending on case location) will send an invite letter through the post to these potentially eligible index cases. The invite letter outlines the study, describes why the case has been contacted, and explains that a research nurse may be in touch over the coming weeks to discuss the study. The letter allows the case to choose several ways of opting-out of this contact (e-mail, freepost, telephone) and provides a named, clinical study lead for each public health organisation should they wish to discuss any aspect of this.

Approaching to recruit

If a contacted index case does not opt-out within two weeks, their details will be shared securely (using internally agreed practices) with the NHS research nurses at the Clinical Research Network North West Coast (CRN). The research nurses will attempt to contact the index case (or parent/guardian of) via telephone (using internally agreed practices) to inform them about the study and offer them the opportunity to participate, if eligible. A maximum of

For peer review only

332 three attempts will be made, and nurses will not leave voicemails. If a case is unable to be
333 contacted, or does not wish to participate at this stage, their details will be deleted from the
334 line list. If the approached index case is successfully contacted via telephone and interested
335 in participating, or would like more information, the research nurses will prepare and post a
336 study pack. Index cases may be excluded at this stage where discussions with the case
337 reveal that any of the following exclusion criteria apply:

338 • Index case is in a single-person household;
339 • Index case is a visitor to a household in the study area, but is registered with a GP
340 outside the study area;
341 • Household is outside the study area;
342 • The index case is resident in an institution: retirement home, nursing home, prison,
343 barracks, boarding school, or college/university halls of residence.

344 **Study packs**

345 The study packs contain:

346 • A study information pamphlet;
347 • A questionnaire booklet for the index case or a suitable representative (e.g. parent,
348 head of household) to complete, with a freepost envelope;
349 • A consent form for each participating household member to read, initial, and sign
350 (forms part of the questionnaire));
351 • A stool sampling pack (Fe-Col®) for each participating household member, with the
352 required return postal envelope;
353 • An information leaflet on cryptosporidiosis and the relevant health advice;
354 • An information sheet on General Data Protection Regulation (GDPR) for health and
355 care research.

356 **Consent**

For peer review only

The index case and any household members who wish to take part will sign and return the consent form at the front of the questionnaire. The return of study materials such as a completed questionnaire and/or stool samples will be considered implied consent.

Disenrollment

If study materials are not received within 14 days of posting the pack, a reminder letter will be dispatched by the research nurses at the CRN. If study documentation is not returned within 14 days of posting the reminder letter, no further attempt at contact will be made and the index case will be removed from the study line list.

Participation

If the household wants to take part, all interested members will sign and return the consent form. At least one household member, as well as the index case, must consent.

Consent forms and questionnaires are returned to the University of Liverpool in a stamped addressed envelope provided in the pack. The unique study IDs of those consenting will be shared weekly with the research nurses at the CRN to cross-match those contacted index cases that have been recruited and enrolled. Each consenting household member will also be asked to provide a stool sample for testing, using the provided Fe-Col® kits, which include a pre-addressed and secure postal bag (compliant with UN3373 regulations for mailing Cat B biological samples [35]). Instructions are provided, and samples will be returned directly to the *Cryptosporidium* Reference Unit.

Data management and oversight

Documentation

Questionnaire data will be inputted from the paper format to a corresponding MS Access database and held securely on a University of Liverpool drive in accordance with their security protocols. Figure 2 shows the data flow expected. Double data entry will be undertaken on a sample of questionnaires and discrepancies resolved using internal validation checks. When data entry is complete the data will be exported to the final study database [MS Access] where the data are pseudonymised for analyses: Name, date of birth,

For peer review only

and full postcode will be removed and replaced with unique study ID, age, and Lower Super Output Area (LSOAs are a type of geographic area in England and Wales, comprised of >1,000 residents [36]).

All data storage, cleaning and analyses will be undertaken by the study team at the University of Liverpool. Data will be stored on institutional network drives with appropriate security measures in place. Hard copy records will be stored in a locked cabinet in a secure location and access to records and data are limited to study personnel. Paper documents will be stored separately from the corresponding electronic data. The sponsor and data controller for this study is the Clinical Governance Team at the University of Liverpool.

Data security protocol available on request

Stool sample management

For purposes of data confidentiality and governance, stool samples returned to the *Cryptosporidium* Reference Unit are pseudonymised with the unique study ID, and participants will be asked to write their age, sex, and date of sample on their sample pot before they collect the stool. Stool sample results will be added to a study-specific database held at the CRU. The stool sample results will be added to the study database at the end of data collection using unique study ID in a secure file transfer.

Identifying the index case samples

Original diagnostic laboratory numbers will be retained with the index case information in the original line lists at PHE/PHW so that the sample can be identified later at the CRU and grouped with the relevant household samples. This sample, when located at the CRU, will be processed in the same way as other study samples.

Full laboratory protocol available on request

Outcomes and measurements

Questionnaire data

The questionnaire is divided into sections and is mostly composed of dichotomous and multiple-choice questions. Section A asks questions to determine the composition of the

For peer review only

household, the clinical details of the index case, and captures any other symptomatic household members. A table is used to collect information on any other symptomatic diarrhoeal illness in the house and will attempt to capture relationships to the index case (Table 3:).

Age	*Sex*	*Relationship to first case*	*Been ill with diarrhoea and/or vomiting*<br><br>*[Yes/No/Don't know]*	*When they became ill*<br><br>*[Date if known, otherwise before/after the first case]*	*How many days were they ill with these symptoms?*	*Did they see a doctor about this illness?*
39	*F*	*Mother*	*Yes*	*18/12/2017*	*10*	*No*
42	*M*	*Father*	*Yes*	*Before*	*About 3 days*	*No*
6 months	*M*	*Brother*	*No*	-		
24	*F*	*Lodger/House mate*	*Don't know*	-		

Table 3: An example table showing information collected in the questionnaire about other illness in the house

Section B records activities of the index case, and others in the home, in the two weeks prior to the index case's onset, based on known exposures for *Cryptosporidium*. Information on outdoor and leisure activities may help determine possible co-primary infections and distinguish them from those that are secondary. (Table 4:)

Sections C and D collect household variables, including the number of bedrooms and bathrooms, and capturing those who share beds/baths, and asking about outside space and animals. We also ask about nappy changing and toilet training in the home, and about general hand-washing behaviour.

Full questionnaire available on request.

For peer review only

Activity	FIRST CASE	ANYONE ELSE IN THE HOME
Travel outside the UK		
Swimming – outdoors in a lake, river, stream etc. (wild swimming)		
Swimming – in a treated swimming pool, either indoors or outdoors (such as a pool at a leisure centre or a lido)		
Other water activities/sport (such as surfing, rowing, water-skiing etc.)		
Other outdoor activities (such as camping, climbing, hiking, cycling etc.)		
Gardening (at home or elsewhere, such as an allotment)		
Contact with pets (at home or with pets at another house)		
Visiting or working on a farm or had contact with farm animals		
Visiting or working at a zoo or had contact with zoo or wild animals		

Table 4: An example table showing information collected in the questionnaire about activities

Stool collection and genotyping

All consenting household members of the index case (but not the index case) will be asked to provide a stool sample using the Fe-Col® kit (Figure 3) provided in the study pack, and post to the Cryptosporidium Reference Unit.

The stool pots will be labelled with the unique household ID which identifies them as part of the study but allows the samples to remain anonymous to the reference laboratory team. Samples will be scored against the Bristol stool scale and tested and quantified, only for *Cryptosporidium*, using immunofluorescence (IF) microscopy and real-time PCR. Positive samples will be speciated using validated PCR techniques which are part of normal practice [37]. *Cryptosporidium* DNA will be retained for subtyping and possibly whole genome sequencing (WGS) at a later date.

For peer review only

Full laboratory protocol available on request

ANALYSES

The primary objective of this study is to determine the amount of spread that happens in the home where there is a case of *Cryptosporidium*. This will be established by testing stool samples of household members of a case for *Cryptosporidium* and reporting the numbers of other cases (according to our pre-determined definitions in Table 1). A household with more than one case of any type will be a household with transmission. As we are only able to capture cases of *Cryptosporidium* using laboratory confirmed cases reported to surveillance, we recognise that what we may define as an 'index' case, may not be, in true epidemiological terms, the first case that has driven transmission. Whilst it is important that cases are identified and recruited based on the same diagnostic criteria, we accept that the identification of index cases is a pre-enrolment definition. Following enrolment of the household into the study, and the return of documentation, an index case may be categorised differently, and may actually fit the definition for a secondary case. This will be analysed at the household level and depends on the accurate population of fields in the questionnaire. In doing this, we are able to more accurately describe transmission in the home, and this may well allow us to describe the characteristics of these true index cases, and why we do not pick them up in surveillance, for example, if they exhibit different health-seeking behaviour.

We will calculate the following:

- The secondary transmission rate/prevalence within households (number of cases in the home/numbers at risk in the home, number of households with secondary spread /number of households));
- The amount of asymptomatic carriage among those exposed to symptomatic cases (number of asymptomatic cases/number at risk));
- Odds/Risk (OR/RR) of secondary illness according to activities and case/household characteristics;

For peer review only

472 • Odds/Risk (OR/RR) of secondary illness according to organism species.

473 Confounding (e.g. host factors such as age, co-morbidity) will be considered, where known, using multivariable techniques. Also, we will, where possible, examine environmental level exposures using stratification, e.g. those households/cases which are exposed to other known sources or risk factors, such as those living on farms.

477 Data will be analysed using Stata v12.

479 **LIMITATIONS AND BIASES**

480 Some elements of the study design are retrospective in nature, as the index case must have already been ill and been tested in order to be selected. As a result, some ascertainment bias may lead to a skewed sample from which to choose the index cases. We do not expect to capture the full profile of cases and households in the population that might have *Cryptosporidium* due to differences in risk or vulnerability, severity, or health-seeking behaviour[38]. We may get an over-representation of severe disease as these cases are more likely to seek health care and be tested, and perhaps more likely to test positive.

487 We are only collecting one sample from each household member, and not re-sampling the index case, for time and resource reasons. This may well lead to missing intermittent shedding of oocysts, tertiary household infections and/or misclassifying recurring illness.

490 As *Cryptosporidium* is common in younger age groups, we expect a large proportion of the participants to represent families with young children which may lead to over-representation of these households. In addition, we might expect that having young children who were ill, or being severely ill themselves, may incentivise cases to participate in the study, more than adult, less severe cases.

495 Any likely over- or under-representation in the data collected will be considered when assessing and describing results. Further unidentifiable limitations may include recall biases around dates of onset or activities, and classification biases as we are asking about self-reported illness and information may be inaccurate.

For peer review only

There is a possibility that we could see ongoing outbreaks in the study year. If this happens, we might try to identify these where possible and may consider excluding households from this study where all or most members have been exposed, including a definition of a co-primary case.

End of study

The study will be declared as ended when the database is closed to recruitment – after one year or when the maximum number of households has been enrolled.

Pilot arrangements

A pilot phase of one month is anticipated before data collection begins to assess and evaluate processes. Pilot data will be included as study data if no major methodological changes are proposed.

Ethics and dissemination

The study has been approved by the North West – Liverpool East NHS Research Ethics Committee (REC Reference: 18/NW/0300) and the Confidentiality and Advisory Group (Reference 18/CAG/0084). The project is registered on the National Institute for Health Research (NIHR) portfolio (CPMS ID – 39458).

Outputs will include scientific conferences and peer-reviewed publications. In addition, a short, lay report of findings will be produced for participants, who can opt to receive this when they take part.

Figure legend/caption

Fig 1: Outline of study

Fig 2: Study flow diagram

Fig 3: Fe-Col kit

References

1 Chalmers RM, Smith R, Elwin K, *et al.* Epidemiology of anthroponotic and zoonotic

For peer review only

human cryptosporidiosis in England and Wales, 2004 to 2006. *Epidemiol Infect* 2011;**139**:700–12.http://www.jstor.org/stable/27975646

2 Cacciò SM, Putignani L. Epidemiology of Human Cryptosporidiosis. In: *Cryptosporidium: parasite and disease*. Vienna: : Springer Vienna 2014. 43–79. doi:10.1007/978-3-7091-1562-6_2

3 Adak GK, Long SM, O'Brien SJ. Trends in indigenous foodborne disease and deaths, England and Wales: 1992 to 2000. *Gut* 2002;**51**:832–41.http://www.ncbi.nlm.nih.gov/pubmed/12427786 (accessed 12 Jun 2017).

4 Food Standards Agency. A report of infectious intestinal disease in England. London: 2000.

5 Cacciò SM, Thompson RCA, McLauchlin J, *et al.* Unravelling Cryptosporidium and Giardia epidemiology. *Trends Parasitol* 2005;**21**:430–7. doi:10.1016/j.pt.2005.06.013

6 Chalmers RM, Giles M. Zoonotic cryptosporidiosis in the UK – challenges for control. *J Appl Microbiol* 2010;**109**:1487–97. doi:10.1111/j.1365-2672.2010.04764.x

7 Hannah J, Riordan T. Case to case spread of cryptosporidiosis; evidence from a day nursery outbreak. *Public Health* 1988;**102**:539–44.https://www.ncbi.nlm.nih.gov/pubmed/3231695

8 Garvey P, McKeown P. EPIDEMIOLOGY OF HUMAN CRYPTOSPORIDIOSIS IN IRELAND, 2004-2006: ANALYSIS OF NATIONAL NOTIFICATION DATA. *EUROSURVEILLANCE* 2009;**14**:16–20.

9 Abubakar I, Aliyu S, Arumugam C, *et al.* Risk factors for sporadic cryptosporidiosis among immunocompetent persons in the United States from 1999 to 2001. *J Clin Microbiol* 2004;**37**:2944–51. doi:10.3201/eid0905.020678

10 Hunter PR, Hughes S, Woodhouse S, *et al.* Sporadic cryptosporidiosis case-control study with genotyping. *Emerg Infect Dis* 2004;**10**:1241–9. doi:10.3201/eid1007.030582

11 Waldram A, Vivancos R, Hartley C, *et al.* Prevalence of Giardia infection in households of Giardia cases and risk factors for household transmission. *BMC Infect*

For peer review only

Dis 2017;**17**:486. doi:10.1186/s12879-017-2586-3

12 Minetti C, Lamden K, Durband C, *et al.* Case-Control Study of Risk Factors for Sporadic Giardiasis and Parasite Assemblages in North West England. *J Clin Microbiol* 2015;**53**:3133–40. doi:10.1128/JCM.00715-15

13 Davies A, Campbell B, Evans MR, *et al.* Asymptomatic carriage of protozoan parasites in children in day care centers in the United Kingdom. *Pediatr Infect Dis J* 2009;**28**:838–40.https://insights.ovid.com/pubmed?pmid=19684527

14 Johansen OH, Hanevik K, Thrana F, *et al.* Symptomatic and asymptomatic secondary transmission of Cryptosporidium parvum following two related outbreaks in schoolchildren. *Epidemiol Infect* 2014;**143**:1–8. doi:10.1017/S095026881400243X

15 Hunter P, Thompson R. The zoonotic transmission of Giardia and Cryptosporidium. *Int J Parasitol* 2005;**35**:1181–90. doi:10.1016/j.ijpara.2005.07.009

16 Frost FJ, Roberts M, Kunde TR, *et al.* How Clean Must Our Drinking Water Be: The Importance of Protective Immunity. *J Infect Dis* 2005;**191**:809–14. doi:10.1086/427561

17 Newman RD, Zu SX, Wuhib T, *et al.* Household epidemiology of Cryptosporidium parvum infection in an urban community in Northeast Brazil. *Ann Intern Med* 1994;**120**:500–5.http://www.ncbi.nlm.nih.gov/pubmed/8311373 (accessed 27 Apr 2017).

18 Korpe PS, Haque R, Gilchrist C, *et al.* Natural History of Cryptosporidiosis in a Longitudinal Study of Slum-Dwelling Bangladeshi Children: Association with Severe Malnutrition. *PLoS Negl Trop Dis* 2016;**10**:e0004564. doi:10.1371/journal.pntd.0004564

19 Korpe PS, Gilchrist C, Burkey C, *et al.* Case-Control Study of Cryptosporidium Transmission in Bangladeshi Households. *Clin Infect Dis* Published Online First: 6 September 2018. doi:10.1093/cid/ciy593

20 ONS. 2011 Census - Office for National Statistics. https://www.ons.gov.uk/peoplepopulationandcommunity/populationandmigration/popul

For peer review only

ationestimates/bulletins/populationandhouseholdestimatesfortheunitedkingdom/2011-03-21#number-of-people-in-households (accessed 11 Jun 2018).

21 PHE. Cryptosporidium: national laboratory data - GOV.UK. https://www.gov.uk/government/publications/cryptosporidium-national-laboratory-data (accessed 11 Jun 2018).

22 Welsh Government | Census of population. Stat. Res. 2015.https://gov.wales/statistics-and-research/census-population/?lang=en (accessed 10 Aug 2018).

23 Wall PG, de Louvois J, Gilbert RJ, *et al.* Food poisoning: notifications, laboratory reports, and outbreaks--where do the statistics come from and what do they mean? *Commun Dis Rep CDR Rev* 1996;**6**:R93-100.http://www.ncbi.nlm.nih.gov/pubmed/8680502 (accessed 10 Aug 2018).

24 Public Health (Control of Disease) Act 1984. 1984.https://www.legislation.gov.uk/ukpga/1984/22 (accessed 10 Aug 2018).

25 The Health Protection (Notification) Regulations 2010. 2010.http://www.legislation.gov.uk/uksi/2010/659/regulation/4/made (accessed 10 Aug 2018).

26 Manuel R, Roche A, Young Y, *et al.* An audit of the laboratory diagnosis of cryptosporidiosis in England and Wales. *J Med Microbiol* 2015;**64**:688–93. doi:10.1099/jmm.0.000089

27 Public Health England. Cryptosporidium reference unit (CRU) - GOV.UK. https://www.gov.uk/guidance/cryptosporidium-reference-unit-cru (accessed 10 Aug 2018).

28 Tam CC, Rodrigues LC, Viviani L, *et al.* Longitudinal study of infectious intestinal disease in the UK (IID2 study): incidence in the community and presenting to general practice. *Gut* 2012;**61**:69–77. doi:10.1136/gut.2011.238386

29 Perry S, De La Luz Sanchez M, Hurst PK, *et al.* Household transmission of gastroenteritis. *Emerg Infect Dis* 2005;**11**:1093–6. doi:10.3201/eid1107.040889

For peer review only

30 Harrison SL, Nelder R, Hayek L, *et al.* Managing a large outbreak of cryptosporidiosis: how to investigate and when to decide to lift a 'boil water' notice. *Commun Dis public Heal* 2002;**5**:230–9.

31 MacKenzie WR, Schell WL, Blair KA, *et al.* Massive outbreak of waterborne cryptosporidium infection in Milwaukee, Wisconsin: recurrence of illness and risk of secondary transmission. *Clin Infect Dis* 1995;**21**:57–62.NS - (accessed 27 Apr 2017).

32 Public health control and management of hepatitis A 2017 Guidelines. Published Online First: 2017.https://www.gov.uk/government/uploads/system/uploads/attachment_data/file/623036/Public_health_control_and_management_of_hepatitis_A_2017.pdf (accessed 30 Oct 2017).

33 Willison DJ, Swinton M, Schwartz L, *et al.* Alternatives to project-specific consent for access to personal information for health research: insights from a public dialogue. *BMC Med Ethics* 2008;**9**:18. doi:10.1186/1472-6939-9-18

34 Taylor MJ, Taylor N. Health research access to personal confidential data in England and Wales: assessing any gap in public attitude between preferable and acceptable models of consent. *Life Sci Soc policy* 2014;**10**:15. doi:10.1186/s40504-014-0015-6

35 Category B - Medical Packaging, medical pouches and biological substances on un3373.com. http://www.un3373.com/category-biological-substances/category-b/ (accessed 10 Aug 2018).

36 Office for National Statistics. Overview of the Various Geographies Used in the Production of Statistics Collected via the UK Census.

37 Robinson G EK and CR. Methods and Protocols in Cryptosporidium Research. Cryptosporidium diagnostic assays: molecular detection. In: Ed. Mead J, ed. *Methods in Molecular Biology*. Springer Nature

38 Adams NL, Rose TC, Hawker J, *et al.* Socioeconomic status and infectious intestinal disease in the community: a longitudinal study (IID2 study). *Eur J Public Health* 2018;**28**:134–8. doi:10.1093/eurpub/ckx091

For peer review only

Authors' contributions

CMCK, RCha, RV, RChr, SOB conceived the initial idea for the study and the methodology. JM contributed to methods for participant recruitment and ethics submissions. CMCK wrote the protocol. RChr, RCha, RV, SOB reviewed and revised the protocol and the paper. All authors have read and approved the final manuscript.

Funding statement

This research is jointly funded by the National Institute for Health Research Health Protection Research Unit (NIHR HPRU) in Emerging and Zoonotic Infections (HPRU EZI) and the National Institute for Health Research Health Protection Research Unit (NIHR HPRU) in Gastrointestinal Infections (HPRU GI) at the University of Liverpool in partnership with Public Health England (PHE), University of East Anglia, University of Oxford and the Institute of Food Research. The views expressed are those of the authors and not necessarily those of the NHS, the NIHR, the Department of Health, PHE or Public Health Wales.

Competing interests statement

The authors declare that they have no competing interests.

Acknowledgements

John Harris, Goutam Adak, Ken Lamden, Andrew Fox

The Clinical Research Network North West Coast, Liverpool

Laura Evans, Public Health Wales

Christopher Williams and the Health Protection Team, Public Health Wales

Jo Hardstaff, University of Liverpool

Richard Dunn and Elaine Mooney, Field Services, Public Health England

Infection and Global Heath panel (PPI group) at the University of Liverpool, Health and Care Research Wales, and all other public/lay contributors to the Public and Patient Involvement survey

For peer review only

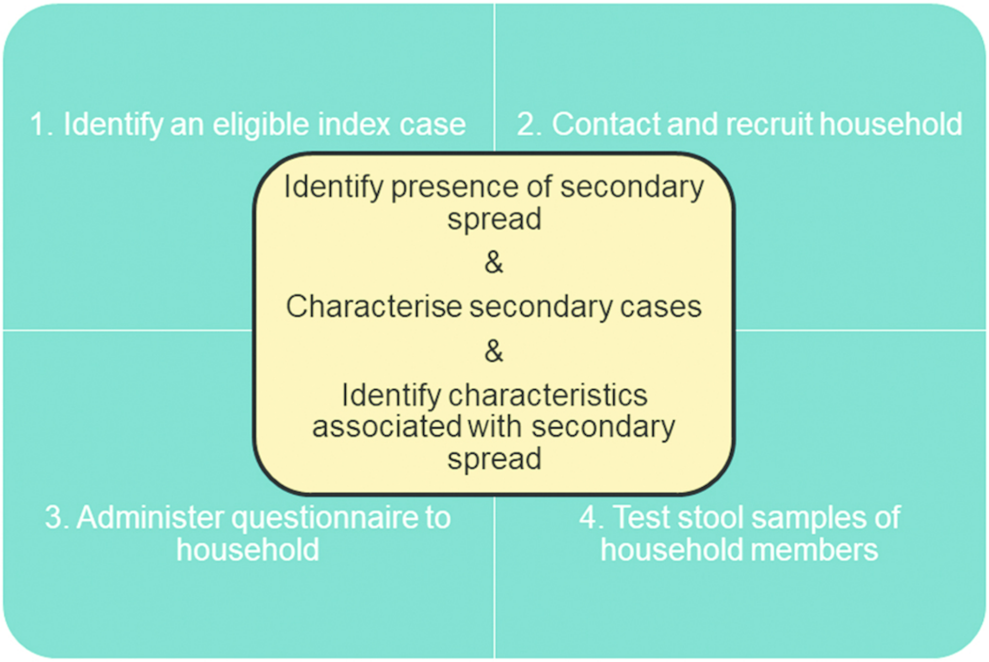

Outline of study

90x90mm (300 x 300 DPI)

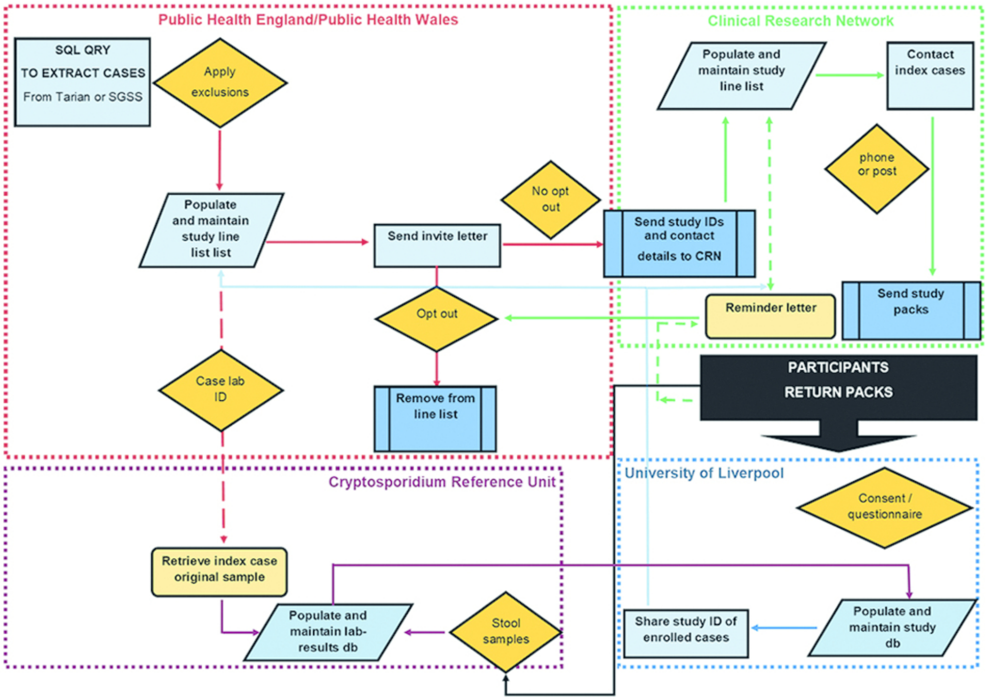

Study Flow Diagram

90x90mm (300 x 300 DPI)

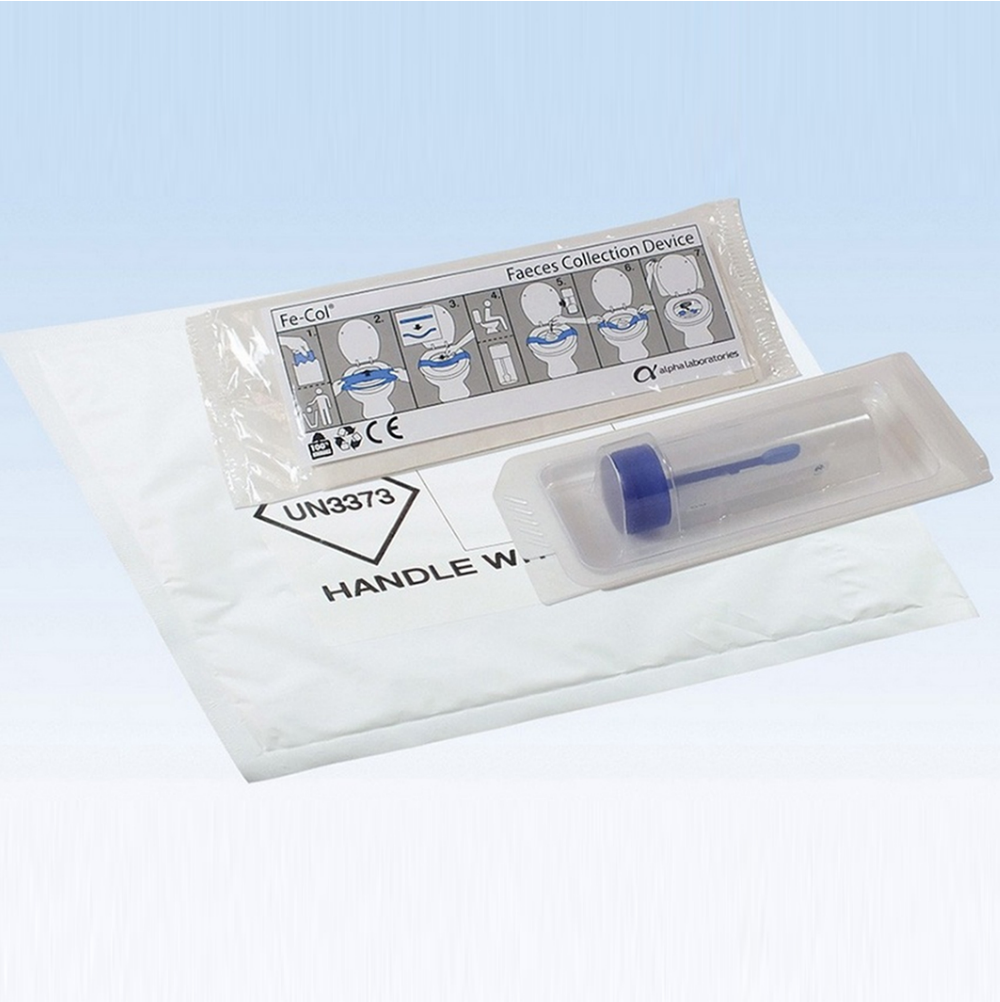

Fe-Col kit

90x90mm (300 x 300 DPI)

STROBE Statement—checklist of items that should be included in reports of observational studies

	Item No	Recommendation
**Title and abstract**	1	(*a*) Indicate the study's design with a commonly used term in the title or the abstract
		(*b*) Provide in the abstract an informative and balanced summary of what was done and what was found
**Introduction**		
Background/rationale	2	Explain the scientific background and rationale for the investigation being reported
Objectives	3	State specific objectives, including any prespecified hypotheses
**Methods**		
Study design	4	Present key elements of study design early in the paper
Setting	5	Describe the setting, locations, and relevant dates, including periods of recruitment, exposure, follow-up, and data collection
Participants	6	(*a*) *Cohort study*—Give the eligibility criteria, and the sources and methods of selection of participants. Describe methods of follow-up<br>*Case-control study*—Give the eligibility criteria, and the sources and methods of case ascertainment and control selection. Give the rationale for the choice of cases and controls<br>*Cross-sectional study*—Give the eligibility criteria, and the sources and methods of selection of participants
		(*b*) *Cohort study*—For matched studies, give matching criteria and number of exposed and unexposed<br>*Case-control study*—For matched studies, give matching criteria and the number of controls per case
Variables	7	Clearly define all outcomes, exposures, predictors, potential confounders, and effect modifiers. Give diagnostic criteria, if applicable
Data sources/ measurement	8*	For each variable of interest, give sources of data and details of methods of assessment (measurement). Describe comparability of assessment methods if there is more than one group
Bias	9	Describe any efforts to address potential sources of bias
Study size	10	Explain how the study size was arrived at
Quantitative variables	11	Explain how quantitative variables were handled in the analyses. If applicable, describe which groupings were chosen and why
Statistical methods	12	(*a*) Describe all statistical methods, including those used to control for confounding we are calculating ORs and percentages. Have outlined objectives.
		(*b*) Describe any methods used to examine subgroups and interactions
		(*c*) Explain how missing data were addressed
		(*d*) *Cohort study*—If applicable, explain how loss to follow-up was addressed<br>*Case-control study*—If applicable, explain how matching of cases and controls was addressed<br>*Cross-sectional study*—If applicable, describe analytical methods taking account of sampling strategy
		(*e*) Describe any sensitivity analyses

Continued on next page

For peer review only - http://bmjopen.bmj.com/site/about/guidelines.xhtml

Results n/a

Participants	13*	(a) Report numbers of individuals at each stage of study—eg numbers potentially eligible, examined for eligibility, confirmed eligible, included in the study, completing follow-up, and analysed
		(b) Give reasons for non-participation at each stage
		(c) Consider use of a flow diagram
Descriptive data	14*	(a) Give characteristics of study participants (eg demographic, clinical, social) and information on exposures and potential confounders
		(b) Indicate number of participants with missing data for each variable of interest
		(c) *Cohort study*—Summarise follow-up time (eg, average and total amount)
Outcome data	15*	*Cohort study*—Report numbers of outcome events or summary measures over time
		Case-control study—Report numbers in each exposure category, or summary measures of exposure
		Cross-sectional study—Report numbers of outcome events or summary measures
Main results	16	(*a*) Give unadjusted estimates and, if applicable, confounder-adjusted estimates and their precision (eg, 95% confidence interval). Make clear which confounders were adjusted for and why they were included
		(*b*) Report category boundaries when continuous variables were categorized
		(*c*) If relevant, consider translating estimates of relative risk into absolute risk for a meaningful time period
Other analyses	17	Report other analyses done—eg analyses of subgroups and interactions, and sensitivity analyses
**Discussion**		
Key results	18	Summarise key results with reference to study objectives
Limitations	19	Discuss limitations of the study, taking into account sources of potential bias or imprecision. Discuss both direction and magnitude of any potential bias
Interpretation	20	Give a cautious overall interpretation of results considering objectives, limitations, multiplicity of analyses, results from similar studies, and other relevant evidence
Generalisability	21	Discuss the generalisability (external validity) of the study results
**Other information**		
Funding	22	Give the source of funding and the role of the funders for the present study and, if applicable, for the original study on which the present article is based

*Give information separately for cases and controls in case-control studies and, if applicable, for exposed and unexposed groups in cohort and cross-sectional studies.

Note: An Explanation and Elaboration article discusses each checklist item and gives methodological background and published examples of transparent reporting. The STROBE checklist is best used in conjunction with this article (freely available on the Web sites of PLoS Medicine at http://www.plosmedicine.org/, Annals of Internal Medicine at http://www.annals.org/, and Epidemiology at http://www.epidem.com/). Information on the STROBE Initiative is available at www.strobe-statement.org.

For peer review only

For peer review only - http://bmjopen.bmj.com/site/about/guidelines.xhtml
